# Comparison of Density Functional and Correlated Wave Function Methods for the Prediction of Cu(II) Hyperfine Coupling Constants

**DOI:** 10.1002/cphc.202000649

**Published:** 2020-11-17

**Authors:** Rogelio J. Gómez‐Piñeiro, Dimitrios A. Pantazis, Maylis Orio

**Affiliations:** ^1^ Aix-Marseille Université CNRS, iSm2 Marseille France; ^2^ Max-Planck-Institut für Kohlenforschung Kaiser-Wilhelm-Platz 1 45470 Mülheim an der Ruhr Germany

**Keywords:** copper, electronic structure, EPR spectroscopy, hyperfine interaction, quantum chemistry

## Abstract

The reliable prediction of Cu(II) hyperfine coupling constants remains a challenge for quantum chemistry. Until recently only density functional theory (DFT) could target this property for systems of realistic size. However, wave function based methods become increasingly applicable. In the present work, we define a large set of Cu(II) complexes with experimentally known hyperfine coupling constants and use it to investigate the performance of modern quantum chemical methods for the prediction of this challenging spectroscopic parameter. DFT methods are evaluated against orbital‐optimized second‐order Møller‐Plesset (OO‐MP2) theory and coupled cluster calculations including singles and doubles excitations, driven by the domain‐based local pair natural orbital approach (DLPNO‐CCSD). Special attention is paid to the definition of a basis set that converges adequately toward the basis set limit for the given property for all methods considered in this study, and a specifically optimized basis set is proposed for this purpose. The results suggest that wave function based methods can supplant but do not outcompete DFT for the calculation of Cu(II) hyperfine coupling constants. Mainstream hybrid functionals such as B3PW91 remain on average the best choice.

## Graphical abstract

**Modern correlated wave function methods** (DLPNO‐CCSD and OO‐MP2) contend against density functional theory for the prediction of Cu(II) hyperfine coupling constants in a test set of realistic copper complexes.

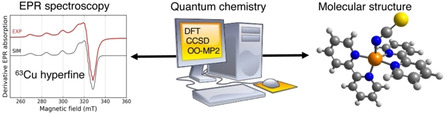

## 1 Introduction

The redox behavior of copper is important for both synthetic chemistry and biology.[^1^, ^2^, ^3^] In particular, the sensitive dependence of the balance between Cu(I) and Cu(II) on the coordination environment, and the geometric variability of the latter in the case of Cu(II), underpin the critical roles of copper ions in catalysis and electron transfer.[^4^, ^5^] Electron paramagnetic resonance (EPR) spectroscopy is the most prevalent method for probing the local electronic structure of Cu(II) centers.[^6^, ^7^, ^8^] The information provided by EPR in the case of mononuclear Cu(II) systems is encoded in the **g** and **A** tensors, which describe respectively the interaction of the unpaired electron density with the external magnetic field (Zeeman interaction) and with the nuclear spin of copper (hyperfine interaction).[^9^, ^10^] Accurate and reliable calculation of these spin Hamiltonian parameters for Cu(II) systems represents a formidable challenge for quantum chemistry.[^11^, ^12^, ^13^] This is especially true for the hyperfine coupling constants (HFCs), *A*, for which three different contributions with distinct physical origins need to be predicted with similar accuracy:

- the Fermi contact term (*A* ^FC^), a first order property which is proportional to the spin density at the nucleus and represents an isotropic contribution to *A*.
- the spin‐dipolar term (*A* ^SD^) corresponding to the magnetic dipole‐dipole interaction between electron and nuclear spins, that is also a first order property and of anisotropic nature.
- the spin‐orbit coupling (*A* ^SO^), a second‐order contribution that involves excited states of the same total spin as the ground state, which in turn can be decomposed as follows:
- the isotropic pseudo‐contact term (*A* ^*PC*^),
- the anisotropic second order dipolar tensor (*A* ^*SO,dip*^).

The latter term might be safely ignored for light elements but becomes significant for most 3*d* elements including copper and can be comparable in magnitude to *A* ^FC^ and *A* ^SD^.

The question of what is the optimal theoretical approach for the prediction of Cu(II) hyperfine coupling constants has been taken up by many studies over the years.[^14^, ^15^, ^16^, ^17^, ^18^, ^19^, ^20^] These have typically focused on comparing and evaluating different density functional theory (DFT) approximations, often in conjunction with other important technical parameters such as basis sets and treatment of spin‐orbit coupling (SOC). Invariably, these studies demonstrate a large spread in the quality of DFT results. Although hybrid functionals are usually to be preferred in terms of average errors, unexpected failures are not uncommon and hence there is no clearly superior choice that can guarantee predictably consistent performance.

Increasingly, the calculation of hyperfine coupling constants, or at least of the first‐order components *A* ^FC^ and *A* ^SD^, is becoming accessible to wave function based methods for systems of “realistic” size, that is, for mononuclear Cu(II) complexes incorporating ligand systems with tens of atoms.[^21^, ^22^, ^23^, ^24^] To a large extent this is facilitated by smart algorithmic approximations and cost‐effective implementations in widely available software packages. Two examples that have been discussed in the literature are the orbital‐optimized second‐order Møller‐Plesset perturbation theory (OO‐MP2)[^21^, ^22^] and the domain‐based local pair natural orbital coupled cluster method with single and double excitations (DLPNO‐CCSD).^[23]^ In comparison to DFT, wave function based methods have the potential for more systematic performance and better control of errors. This has been commonly accepted for energetics, and it is assumed that the same holds for properties such as hyperfine coupling constants. However, it is unclear under what conditions this is true, for example at which level of the coupled cluster expansion can the superiority of the wave function method over DFT be taken for granted. It is also unclear whether the limited methodological comparisons available in the computational literature can be safely generalized, since they often use minimalistic model systems and are presented chiefly for demonstrative purposes.

In the present work, we aim to address both of the above points. First, we define a large set of Cu(II) complexes with experimentally known structures and experimentally resolved hyperfine coupling tensors. This complex set exhibits great variability in the intrinsic properties of its members. The coordinating sites around the copper centers comprise either one type of coordinating atom: 4N, 4S, 4O, 5N, 6S, or a combination of these: 2N2S, 2N2O, 3N1O. This ensures that both the electronic properties of the complexes and their coordinating geometries are varied: square planar, distorted square planar, tetrahedral, distorted square base pyramidal, and octahedral. Also, some ligands may exhibit chelating effects, bidentate, tridentate, or tetradentate. These differences in coordination chemistry provide a complete set of small mononuclear Cu(II) complexes. Other systems have been studied such as zeolite cluster models,^[25]^ protein active sites,[^26^, ^27^] or dinuclear Cu(I) complexes with ligand‐radical nature,^[28]^ but these fall outside of the scope of this work in terms of defining a benchmark set. Compared to previous work,[^12^, ^17^] we emphasize that the members of the present test set have been independently curated for both their experimental structures and their EPR parameters. Additionally, several complexes were screened out from the final set reported in the present work because of electronic structure problems arising, for example, due to highly negative total charge. With this set at hand, we proceed to define a crucial methodological parameter that may be a source of unsystematic errors when methods of different theoretical nature are compared, i. e. the basis set to be used for copper in the calculation of hyperfine coupling constants. Subsequently, and in addition to other technical parameters, we compare a series of DFT methods that represent all families of functionals, including double‐hybrid functionals. Finally, we present hyperfine coupling constants for all complexes obtained by several wave function based methods. Our analysis of results and comparison of methods allows us to place the performance of the tested methods into a realistic perspective, to make concrete evaluations of the currently available methods, and to present suggestions for future developments.

## Computational Details

Crystallographic coordinates of all complexes were obtained from the Cambridge Structural Database and were individually edited for completeness, chemical correctness, and to remove solvent molecules and non‐coordinating counter‐ions prior to generation of initial structures that were used in the calculations. All calculations were performed with ORCA.^[29]^ Geometry optimizations were performed with the BP86 functional[^30^, ^31^] and the def2‐TZVP basis sets,^[32]^ in combination with corresponding auxiliary basis sets.^[33]^ Increased integration grids were employed (Grid4 In ORCA nomenclature) and tight energy convergence settings were applied throughout. Vibrational frequencies were computed for all optimized structures and the absence of imaginary modes confirmed that true minima were obtained in all cases.

DFT methods evaluated in the present work include PBE,^[34]^ TPSS,^[35]^ SCAN,^[36]^ B3LYP,[^37^, ^38^] CAM‐B3LYP,^[39]^ B3PW91,[^37^, ^40^] PBE0,^[41]^ TPSSh,^[42]^ M06,^[43]^ M06‐2X,^[43]^ and B2PLYP.^[44]^ Computation of hyperfine coupling constants used increased general integration grids (Grid6 in ORCA convention), further increased radial integration accuracy (IntAcc 6.0) and specially enhanced grids for copper (SpecialGridIntAcc 11). For all ligand atoms, the def2‐TZVP basis sets^[32]^ were used. A large variety of basis sets were tested for Cu and further details will be provided in the next section. No approximations to two‐electron integrals were used when calculating hyperfine coupling constants in order to exclude any potential error related to density fitting approximations. Spin‐orbit contributions were included through mean‐field and effective potential approaches The spin‐orbit coupling operator was treated by an accurate mean‐field (SOMF) approximation to the Breit‐Pauli operator (SOCType 3 in ORCA).[^45^, ^46^] The potential was constructed to include one‐electron terms, compute the Coulomb term in a semi‐numeric way, incorporate exchange via one‐center exact integrals including the spin‐other orbit interaction, and include local DFT correlation (SOCFlags 1,2,3,1 in ORCA). It is worth noting that the Coulomb term can be computed in three ways: 1) semi‐numerically, 2) semi‐numerically using the RI approximation, or 3) exactly.^[45]^ The results with the RI approximation exhibited unreasonable HFC values with hybrid functionals, mainly affecting the spin‐orbit contribution, while the exact results did not show any difference from the semi‐numeric approach except for a longer running time (Table S1). Scalar relativistic calculations were performed with the second‐order Douglas‐Kroll‐Hess (DKH2)[^47^, ^48^, ^49^, ^50^, ^51^, ^52^, ^53^] and the zeroth‐order regular approximation (ZORA)[^54^, ^55^, ^56^] Hamiltonians. Given the different behavior of these methods close to the nucleus,^[57]^ the appropriateness of the basis set for Cu was carefully studied and purpose‐made basis sets were used where available. In the case of DKH2 calculations both a point nucleus and a finite nucleus model with a Gaussian charge distribution^[58]^ were tested. Picture change effects were applied as appropriate. The effect of considering the solvent via a continuum solvation model was investigated and found to be non‐essential (Tables S2–S3).

Wave function methods used in this work include MP2, orbital‐optimized MP2 (OO‐MP2) and its spin‐component scaled version, OO‐SCS‐MP2.[^21^, ^59^, ^60^] Relaxed densities were used for MP2; the densities of OO methods are by definition relaxed densities. DLPNO‐CCSD calculations were initiated with unrestricted Kohn‐Sham orbitals and followed the standard DLPNO procedure.[^61^, ^62^] The thresholds used for the DLPNO‐CCSD calculations of hyperfine coupling constants were all tighter than default cutoffs, in line with previous recommendations for spin densities.^[23]^ In detail, the various settings were as follows: TCutMKN=1.0×10^−4^, TCutDO=5.0×10^−3^, TCutPNO=1.0×10^−7^, TCutPNOSingles=0.00, TScalePNOCore=1.0×10^−3^, and TScalePNOSOMO=1.0×10^−1^. In the wave function based calculations of hyperfine coupling constants, all electrons were set as active. “Unrelaxed” spin densities are used for DLPNO‐CCSD (as defined by Saitow and Neese,^[23]^ i. e. where the effect of orbital relaxation is recovered only through the action of exp(*T*_1_) onto the reference). Only the *A* ^FC^ and *A* ^SD^ components can be computed with these methods. The lack of the *A* ^SO^ component means that final values for comparison with experiment must combine the wave function values of the first‐order components with the second‐order contribution obtained from a different method. The def2‐TZVP basis sets with their corresponding auxiliary basis sets for correlated calculations^[63]^ were used for all light atoms. For Cu we employed our tailor‐made basis set described in the following section and based on the primitives of the aug‐cc‐pVTZ‐J basis set of Hedegård et al.^[64]^ Since our modified basis set (denoted aug‐cc‐pVTZ‐Jmod) does not have a specific auxiliary counterpart, we generated a very large auxiliary basis set with the “AutoAux” procedure of ORCA.^[65]^

## 2 Results and Discussion

### 2.1 Reference Set of Cu(II) Complexes

**Figure 1:**
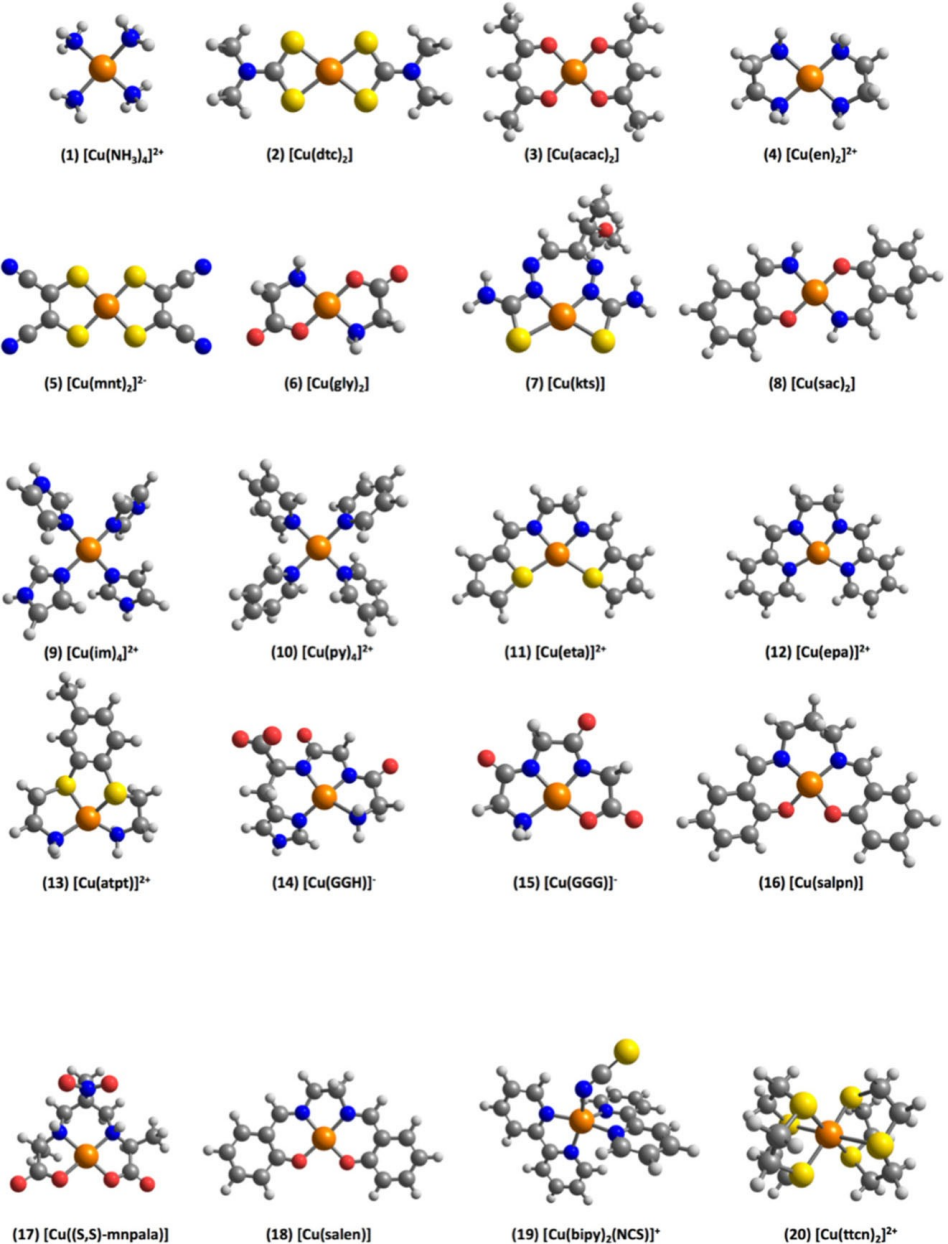
Molecular structures of the Cu(II) complexes considered in the present study. Definition of ligand abbreviations: dtc=dimethyldithiocarbamate, acac=acetylacetone; en=ethylenediamine; mnt=maleonitriledithiolate; gly=glycine; kts=2‐keto‐3‐ethoxybutyraldehyde‐bis(thiosemicarbazone); sac=salicylaldehyde imine; im=imidazole; py=pyridine; eta=N,N’‐ethylenebis(thiophene‐2‐aldimine); epa=N,N’‐ethylenebis(pyridine‐2‐aldimine); atpt=3,4‐bis(3‐amino‐1‐thiopropyl)toluene; GGH=glycine‐glycine‐histidine; GGG=glycine‐glycine‐glycine; salpn=N,N’‐bis(salicylidene)‐1,2‐propanediamine; (S,S)‐mnpala=2,5,8‐trimethyl‐5‐nitro‐3,7‐diazanonanedioate; salen=bis(saliculidene)ethylenediamine; bipy=2,2’‐bipyridine; ttcn=1,4,7‐trithiacyclononane.

Following extensive analysis of the literature, we collected twenty Cu(II) complexes that fulfilled the criteria of being molecular entities with well‐defined crystallographically characterized structure, and having reliable experimentally resolved components of the Cu(II) hyperfine coupling constants. These are depicted in Figure 1, and their experimentally determined hyperfine coupling constants are provided in Table 1. The complexes encompass a wide range of ligands and ligand atom types so that the set is sufficiently varied.

**Table 1:** List of the Cu(II) complexes considered in the present study and their experimentally determined ^63^Cu hyperfine coupling constants (absolute values, in MHz).

Entry	Complex	*A_x_*	*A_y_*	*A_z_*	Ref.
**1**	[Cu(NH_3_)_4_]^2+^	68.8	68.8	586.5	[66]
**2**	[Cu(dtc)_2_]	140.1	140.1	487.3	[67]
**3**	[Cu(acac)_2_]	35.0	35.0	520.0	[68]
**4**	[Cu(en)_2_]^2+^	78.0	82.0	602.0	[69]
**5**	[Cu(mnt)_2_]^2−^	118.0	118.0	500.0	[70]
**6**	[Cu(gly)_2_]	43.1	43.1	529.9	[66]
**7**	[Cu(kts)]	92.9	92.9	575.1	[71]
**8**	[Cu(sac)_2_]	60.0	60.0	470.7	[72]
**9**	[Cu(im)_4_]^2+^	63.2	63.2	563.8	[66]
**10**	[Cu(py)_4_]^2+^	48.8	48.8	563.5	[66]
**11**	[Cu(eta)]^2+^	90.5	90.5	531.8	[73]
**12**	[Cu(epa)(H_2_O)]^2+^	92.9	92.9	524.6	[74]
**13**	[Cu(atpt)]^2+^	54.0	54.0	320.8	[75]
**14**	[Cu(H_‐2_GGH)]^−^	66.0	66.0	620.0	[17]
**15**	[Cu(H_‐2_GGG)]^−^	63.0	63.0	602.6	[17]
**16**	[Cu(salpn)_2_]	47.1	47.1	526.7	[76]
**17**	[Cu(S,S‐mnpala)_2_]	66.0	66.0	581.6	[77]
**18**	[Cu(salen)_2_]	100.4	100.4	613.7	[78]
**19**	[Cu(bipy)_2_(NCS)]^+^	44.1	44.1	468.0	[79]
**20**	[Cu(ttcn)_2_]^2+^	60.0	60.0	458.7	[80]

### 2.2 Definition of the Optimal Cu Basis Set

The choice of basis set for copper is a critical methodological parameter for the calculation of HFCs and needs to be carefully studied before embarking in an extensive comparison of different quantum chemical methods for the complete test set. The nature of the property requires high flexibility of the basis set in the core region, i. e. in the inner Cu *s* orbitals, so that the sensitive variations of spin density on and close to the nucleus are adequately captured. This directly affects the calculation of the Fermi contact term. As the range of *s* functions with high exponents is not relevant for typical chemistry applications that focus on reactivity, standard general‐purpose basis sets are inflexible in the core region and hence inadequate for calculations of hyperfine coupling constants, even if they are large and flexible in the frontier orbital region. Valence basis functions are of course equally important for the calculation of hyperfine coupling constants because they directly affect the spin‐dependent terms. Moreover, high angular momentum functions in the basis set are crucial when correlated wave function methods are employed because they serve as correlation functions.

In the present study, we set from the beginning a number of requirements that a basis set should satisfy. First of all, the basis set must not be treated as an adjustable parameter that can be used to distort the performance of a given method through error compensation, therefore it should provide effectively converged results for all components of the hyperfine coupling constant. Second, the basis set should be robust enough to be used both in a non‐relativistic and in a scalar relativistic framework. Third, it should be sufficiently flexible to provide sufficiently converged results for the target property for both DFT and correlated wave function methods. And fourth, it should remain a generally applicable basis set, i. e. small enough to avoid numerical instabilities in the form of linear dependencies, and with a valence subspace that can be combined in a balanced way with standard basis sets for the ligands. In the following we describe our attempts to define such a basis set.

Two types of basis sets proposed as appropriate for the calculation of core properties include CP(PPP), originally engineered for the calculation of Mössbauer parameters,^[81]^ and aug‐cc‐pVTZ‐J[^64^, ^82^] that specifically targets the calculation of hyperfine coupling constants. A common feature of both is the increased flexibility of the *s* subset and the inclusion of steep *s* functions, albeit with different exponents and overall design as will be discussed in the following. CP(PPP) is a [17s7p3d1f] basis set with a total of 60 contracted functions. It is based on the primitives of the original 1994 Ahlrichs [8s5p3d] VTZ basis set^[83]^ with fully decontracted *s* functions, addition of three tight and closely spaced *s* primitives obtained from the innermost exponent by successive multiplications with a factor of 2.5, and finally the addition of two *p* and one *f* polarization functions. The aug‐cc‐pVTZ‐J basis set of Hedegård et al.^[64]^ is a [17s10p7d3f2g] generally contracted set with 121 functions, using primitives from the original aug‐cc‐pVTZ with the addition of four tight *s*, one *p*, and one *d* functions. Its fully decontracted version, denoted aug‐cc‐pVTZ‐Junc, corresponds to a [25s18p10d3f2g] set with a total of 168 primitive functions (after removing duplicate primitives from the generally contracted functions); this is the largest and most flexible basis set of the present study, and is therefore used as the reference for all others.

Table 2 presents a comparison of these as well as other standard basis sets for the computation of the Cu HFCs of [Cu(NH_3_)_4_]^2+^, obtained with the PBE0 functional. It is worth noting that all results discussed below have been confirmed for two other complexes ([Cu(dtc)_2_] and [Cu(acac)_2_]) using two additional functionals (TPSSh and B3PW91) and are reported in the Supporting Information (Tables S4–S19 and Figures S1‐S8). Several points become immediately apparent from Table 2. First, the Fermi contact term is extremely sensitive to the *s* functions of the basis set. The aug‐cc‐pVTZ‐J (or Junc) and CP(PPP) basis sets are in close agreement with respect to *A* ^FC^ which is due to an important core‐shell spin polarization contribution of negative sign (Table 3). In contrast, the standard aug‐cc‐pVTZ and def2‐TZVP basis sets produce essentially useless results and display positive contributions for core‐shell spin polarization, the 2 *s* contribution being underestimated leading to a poor description of the isotropic term of the Cu HFCs. The situation is identical for the aug‐cc‐pwCVTZ and cc‐pwCVTZ basis sets that contain core‐correlating functions, confirming that the use of basis sets not explicitly adjusted for HFCs should be avoided. For these latter basis sets, the core‐shell spin polarization contributions are again affected with underestimated 2 *s* contributions and leading to low and positive *A_iso_* values. The importance of the flexibility of the *s* subset on the *A* ^FC^ term becomes apparent by testing a version of def2‐TZVP where the *s* functions are simply decontracted (denoted def2‐TZVP‐uncS in Table 2): The Fermi contact term is now drastically improved and the overall differences from the reference values are due to differences in the other terms as much as the *A* ^FC^. Indeed, there is complete remediation of the core‐shell spin polarization contribution, both in sign and magnitude (Table 3). This observation justifies the concept behind the original creation of the CP(PPP) basis sets as well as the use of similarly “core property” modified versions of scalar‐relativistic def2‐TZVP basis sets in practical applications.^[84]^ A second important observation is that CP(PPP) underestimates slightly but non‐negligibly the reference values for *A* ^SD^ and for the largest component of *A* ^SO^. Given that the values of these components are predicted consistently between the fully decontracted reference basis set and others that have a similar coverage of the valence space, this deviation is ascribed to a suboptimal coverage of the *p* subspace in CP(PPP), a deficiency likely inherited from the parent Ahlrichs VTZ basis set.

**Table 2:** Calculated HFCs (individual components and detailed contributions, in MHz) for **[Cu(NH_3_)_4_]^2+^** obtained with the PBE0 functional using different basis sets.

Basis Set	Total			Isotropic		Anisotropic					
	A_11_	A_22_	A_33_	A^FC^	A^PC^	A_11_^SD^	A_22_^SD^	A_33_^SD^	A_11_^SO,dip^	A_22_^SO,dip^	A_33_^SO,dip^
aug‐cc‐pVTZ‐Junc	−26.3	−26.8	−610.3	−360.8	139.7	257.3	256.7	−513.9	−62.4	−62.3	124.7
aug‐cc‐pVTZ‐J	−24.8	−25.2	−607.4	−359.9	140.8	257.2	256.7	−513.9	−62.9	−62.7	125.7
CP(PPP)	−33.2	−33.6	−602.4	−357.8	134.7	249.6	249.1	−498.8	−59.7	−59.6	119.5
aug‐cc‐pVTZ	−276.6	339.6	339.0	−9.7	143.7	−536.7	268.1	268.7	127.2	−63.5	−63.6
aug‐cc‐pwCVTZ	211.2	211.6	−372.4	−122.3	139.1	256.6	257.1	−513.7	−62.2	−62.3	124.5
cc‐pwCVTZ	212.6	213.0	−370.5	−120.5	138.9	256.2	256.8	−513.0	−62.0	−62.1	124.2
def2‐TZVP	−249.5	345.8	346.2	4.8	143.2	−521.6	260.5	261.1	124.6	−62.3	−62.4
def2‐TZVP‐uncS	−3.2	−3.7	−599.1	−345.4	143.3	261.2	260.7	−521.9	−62.4	−62.3	124.9
aug‐cc‐pVTZ‐Jmod	−23.8	−24.3	−606.4	−359.5	139.1	258.0	257.4	−515.4	−62.4	−62.3	124.8
*Exp*.	*68.8*	*68.8*	*586.5*

**Table 3:** Core and valence‐shell spin contributions to the isotropic hfcs (in MHz) for **[Cu(NH_3_)_4_]^2+^** obtained with the PBE0 functional using different basis sets.

Basis Set	**1** ***s***	**2** ***s***	**3** ***s***	Core	Valence	A_iso_	$\left\langle S^{2}\right\rangle$	APD($\left\langle S^{2}\right\rangle$ )
aug‐cc‐pVTZ‐Junc	−17.5	−622.7	437.4	−202.9	−18.3	−221.1	0.7527	0.36
aug‐cc‐pVTZ‐J	−17.9	−622.9	438.7	−202.2	−16.9	−219.1	0.7527	0.36
CP(PPP)	−17.9	−610.1	430.1	−198.0	−25.1	−223.1	0.7526	0.34
aug‐cc‐pVTZ	−19.6	−347.9	506.8	139.3	−5.3	134.0	0.7527	0.36
aug‐cc‐pwCVTZ	−17.5	−433.6	489.9	38.7	−21.9	16.8	0.7527	0.36
cc‐pwCVTZ	−17.4	−433.6	491.5	40.5	−22.1	18.4	0.7527	0.36
def2‐TZVP	−19.3	−361.6	537.8	156.8	−9.3	147.5	0.7527	0.36
def2‐TZVP‐uncS	−24.7	−589.1	423.6	−190.2	−11.8	−202.0	0.7527	0.36
aug‐cc‐pVTZ‐Jmod	−17.9	−620.7	436.3	−202.3	‐15.9	−218.2	0.7527	0.36
					*Exp*.	*241.4*	*0.7500*

The above results advocate the use of the purpose‐made aug‐cc‐pVTZ‐J basis set for the calculation of Cu HFCs, since it produces results indistinguishable from its fully decontracted version. Similar conclusions can be drawn from the results obtained with the [Cu(dtc)_2_] and [Cu(acac)_2_] complexes when using the PBE0, B3PW91 and TPSSh functionals, as reported in the Supporting Information.

To assess the performance of the basis sets, the absolute percent deviations (APD) are evaluated with respect to experimental results for three relevant parameters: *A_33_*, *A_iso_* and *ΔA*, with *A_33_* being the largest component of the hyperfine tensor, *A_iso_* being the average HFC $\left[\left(A_{11}+A_{22}+A_{33}\right)/3\right]$ and *ΔA* representing the anisotropic difference (*A_33_‐A_11_*). The APDs are evaluated in the following manner:

$$APD\left(A_{33}^{\left(M\right)}\right)=\;\left|\frac{A_{33}^{\left(M\right)}-A_{33}^{exp}}{A_{33}^{exp}}\right|\times 100,$$

$$APD\left(A_{iso}^{\left(M\right)}\right)=\;\left|\frac{A_{iso}^{\left(M\right)}-A_{iso}^{exp}}{A_{iso}^{exp}}\right|\times 100,$$

$$\mathrm{where}\;A_{iso}^{\left(M\right),exp}=\;\left(A_{11}^{\left(M\right),exp}+A_{22}^{\left(M\right),exp}+A_{33}^{\left(M\right),\;exp}\right)/3,\;\mathrm{and}$$

$$APD\left({\Delta A}^{\left(M\right)}\right)=\;\left|\frac{{\Delta A}^{\left(M\right)}-{\Delta A}^{exp}}{{\Delta A}^{exp}}\right|\times 100,\mathrm{where}\;{\Delta A}^{\left(M\right),exp}=A_{max}^{\left(M\right),\;exp}-\;A_{min}^{\left(M\right),exp}$$

**Figure 2:**
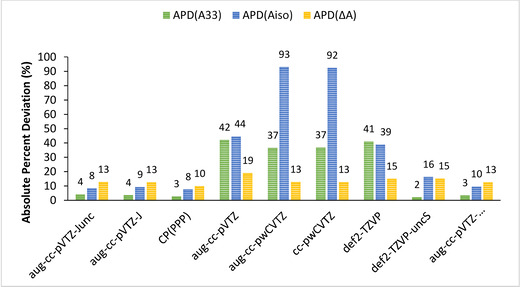
Graphical representation of the APDs of *A_33_*, *A_iso_*, and *ΔA* for calculated values of **[Cu(NH_3_)_4_]^2+^** obtained with the PBE0 functional using different basis sets.

The evaluation of the APD for **[Cu(NH_3_)_4_]^2+^** presented in Table 4 and Figure 2 finally supports the above analysis regarding the necessity of a high flexibility of the basis set in the core region and the relevance of the aug‐cc‐pVTZ‐J (or Junc) basis sets to predict Cu HFCs.

**Table 4:** APDs of *A*_*33*_, *A_iso_*, and *ΔA* for calculated HFCs of **[Cu(NH_3_)_4_]^2+^** obtained with the PBE0 functional using different basis sets and compared with experimental data.

Basis set	A_33_	A_iso_	ΔA	APD(A_33_)	APD(A_iso_)	APD(ΔA)
aug‐cc‐pVTZ‐Junc	−610.3	221.1	584.0	4.1	8.4	12.8
aug‐cc‐pVTZ‐J	−607.4	219.1	582.6	3.6	9.2	12.5
CP(PPP)	−602.4	223.1	569.2	2.7	7.6	9.9
aug‐cc‐pVTZ	339.0	134.0	616.2	42.2	44.5	19.0
aug‐cc‐pwCVTZ	−372.4	16.8	584.0	36.5	93.0	12.8
cc‐pwCVTZ	−370.5	18.4	583.5	36.8	92.4	12.7
def2‐TZVP	346.2	147.5	595.7	41.0	38.9	15.1
def2‐TZVP‐uncS	−599.1	202.0	595.9	2.1	16.3	15.1
aug‐cc‐pVTZ‐Jmod	−606.4	218.2	582.6	3.4	9.6	12.5
*Exp*.	*586.5*	*241.4*	*517.7*

An additional point concerns the robustness of the basis set with respect to scalar relativistic effects, which result in spatial contraction of *s* and *p* orbitals.^[57]^ These data are presented in Supplementary material (Tables S20–S21) and propose a similar comparison for [Cu(NH_3_)_4_]^2+^ using the DKH2 Hamiltonian and appropriate corresponding basis sets. Here again aug‐cc‐pVTZ‐Junc serves as the reference. DKH‐adapted versions of the standard cc‐type basis sets similarly fail for the *A* ^FC^ term, but the DKH‐adapted version^[85]^ of def2‐TZVP,^[32]^ developed to be used in conjunction with the SARC family of all‐electron basis sets,[^85^, ^86^, ^87^, ^88^, ^89^, ^90^] performs very well for all components, owing to the DKH‐optimized contraction coefficients and the partially decontracted *s* and *p* shells. CP(PPP) can be considered adequate for the *A* ^FC^ term but it shows the same type of deviation as seen in the non‐relativistic calculations for both *A* ^SD^ and *A* ^SO^ components. Importantly, the contracted aug‐cc‐pVTZ‐J version deviates from the reference for the crucial Fermi contact term showing that it suffers from the contraction of *s* functions, whereas the *A* ^SD^ and *A* ^SO^ terms are practically the same as the reference values.^[91]^

The finite nucleus model available with the ORCA program package was applied with the DKH2 Hamiltonian for [Cu(NH_3_)_4_]^2+^, employing the PBE0 and B3PW91 functionals (Tables S22–S23). Applying the finite nucleus model has a very limited effect on the computed HFCs. Regardless of the functional used, the DK version of the cc basis sets still underperform and the aug‐cc‐pVTZ‐J keeps deviating from the ‐Junc analogue while predicted HFCs with both CP(PPP) and DKH‐def2‐TZVP are close to those obtained with the reference. Overall, the finite nucleus model has a very limited effect on the calculated HFCs and we recover the main trends obtained without using it.

Identical conclusions are reached when the ZORA Hamiltonian is used instead of DKH2 for the inclusion of scalar relativistic effects (Tables S24‐S25). Considering the results with aug‐cc‐pVTZ‐Junc as reference, the CP(PPP) and the ZORA version of the def2‐TZVP perform identically and provide computed HFCs of similar sign and magnitude. The above observations combined with the evaluation of the APD for both DKH2 and ZORA Hamiltonians **(**Table S26 and Figures S9–S10) finally support that inclusion of scalar relativistic corrections is not statistically crucial for an accurate prediction of Cu HFCs, the limiting parameter here being the definition of appropriate basis sets. While scalar relativity is usually important to model the properties of transition metal complexes, it does not seem to clearly improve the prediction of copper HFCs in an average sense. Therefore, for the sake of simplicity our study does not incorporate scalar relativistic corrections.

A further consideration in the context of the present study is how appropriate a basis set is for use with correlated wave function methods. Simple tests with MP2 and CCSD calculations on [Cu(NH_3_)_4_]^2+^ confirmed the expectation that aug‐cc‐pVTZ‐J and ‐Junc recover massively more correlation energy than the CP(PPP), in line with the absence of appropriate correlation functions in the latter. All the above observations taken together would seem to suggest that the reference ‐Junc basis set would be the safest choice, however this would be computationally cumbersome. Moreover, it is clear from Tables 2 and 3 that decontraction of shells with angular momenta higher than *s* is unnecessary. This led us to investigate modifications of the ‐J basis sets that bring the results as close as possible to the reference while constraining the computational cost. The decontraction of *s* functions assures converged *A* ^FC^ values both in non‐relativistic and scalar relativistic calculations. However, close inspection of the Cu 1s orbital revealed that the three innermost *s* primitives remain practically unused and have near‐zero coefficients. The only condition under which we saw these functions making a noticeable, yet not significant, difference in HFC results was in DKH2 calculations that made use of the finite nucleus model, an approach that will not be further explored here. For the present purposes we therefore define a modified version of aug‐cc‐pVTZ‐J with decontracted *s* functions and removal of the three innermost *s* primitives ([22s10p7d3f2g], denoted aug‐cc‐pVTZ‐Jmod) as a computationally optimal basis set for the calculation of Cu HFCs that is applicable to all methods considered in the present study.

### 2.3 Comparison of DFT Methods

In this section we compare the performances of density functional methods to predict reliable and accurate Cu hyperfine coupling constants. For that purpose, we exclusively employ our modified basis set, aug‐cc‐pVTZ‐Jmod, and conduct calculations on the complete set of copper complexes. Diverse density functionals are considered such as: GGA (PBE), hybrid (PBE0, B3LYP, B3PW91) and range‐separated hybrid (CAM‐B3LYP); meta‐GGA (TPSS, SCAN) and hybrid meta‐GGA (M06, M062X, TPSSh); and double hybrid (B2PLYP). As shown in the previous section, no scalar relativity corrections need to be included in the calculations as they were proven to make no difference on average. On a more technical note, we observed that hybrid functionals are sensitive to the method used for calculating the spin‐orbit contribution. When the second flag of the SOC operator was set to compute the Coulomb term with the RI approximation, the calculated HFCs deviated remarkably from the experimental values. Setting this parameter to compute the Coulomb term exactly leads to good agreement between calculated and experimental results. When this flag was set to default (this study), the Coulomb term was computed semi‐numerically and the obtained hyperfine values are practically indistinguishable from those obtained by exact computation of the Coulomb term.

**Figure 3:**
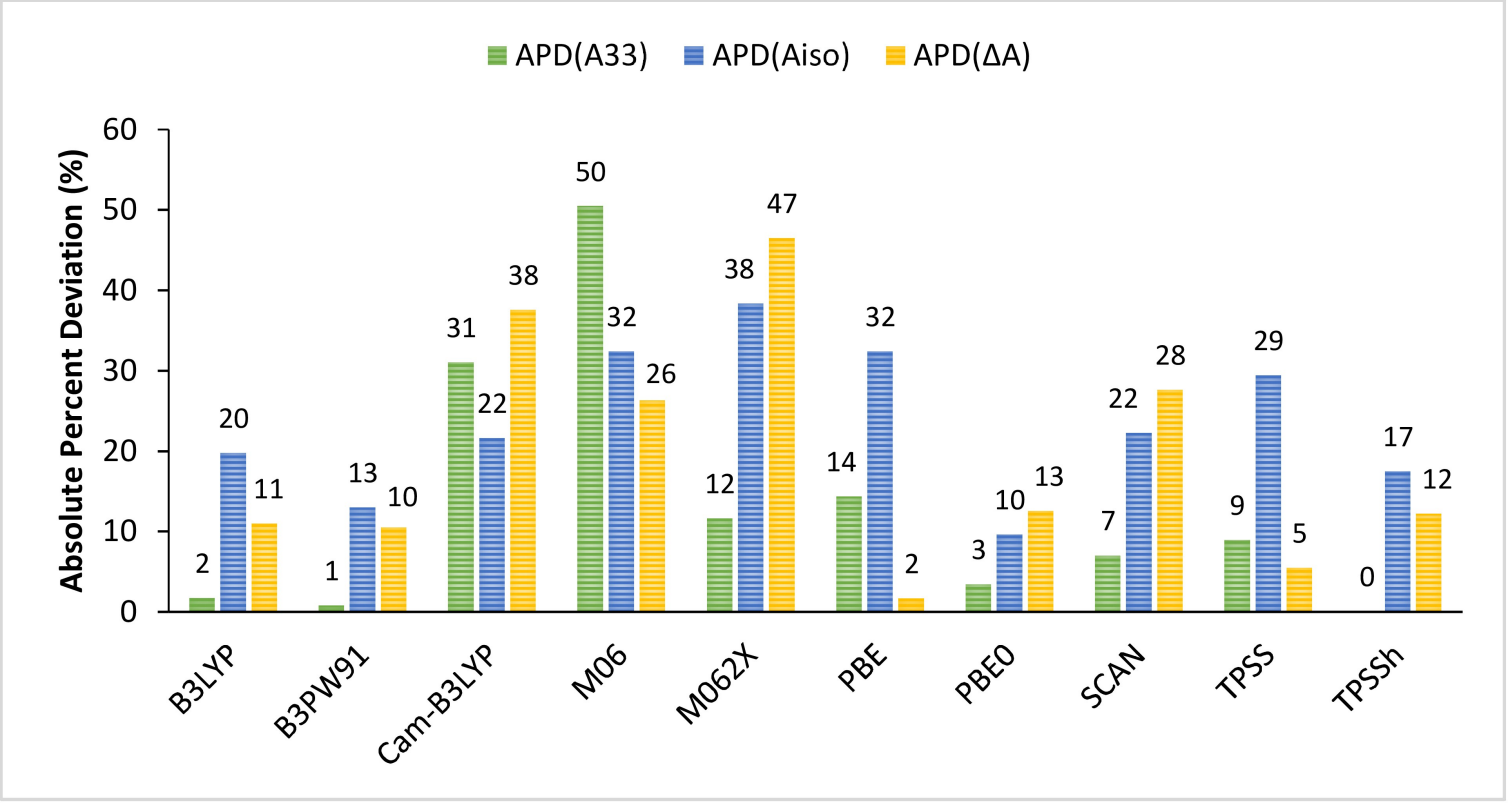
Graphical representation of the APDs of *A*_*33*_, *A_iso_*, and *ΔA* for calculated values of **[Cu(NH_3_)_4_]^2+^** obtained with the aug‐cc‐pVTZ‐Jmod basis using different functionals with respect to experimental results.

Looking at Table 5, we observe that hybrid functionals (PBE0, B3LYP and B3PW91) reproduce quite well the experimental hyperfine coupling constants of the reference complex [Cu(NH_3_)_4_]^2+^. This is not the case for both GGA (PBE), meta‐GGA (TPSS, SCAN) and range‐separated (CAM‐B3LYP) functionals which deviate from experiment. While PBE and TPSS indeed provide low *A_iso_* term due to the underestimation of the core‐shell spin polarization contributions, the case of CAM‐B3LYP is quite different as it suffers from an incorrect description of the spin‐orbit contribution with underestimation from both pseudo‐contact and second‐order dipolar terms. The Minnesota functionals (M06 and M062X) do not reproduce the experimental values at all and behave quite erratically as no trend can be extracted from the predicted individual contributions. These observations are in line with previous studies[^17^, ^92^] which reported the inadequate behavior of the Minnesota functionals due to the incorrect description of core spin‐densities. From Table 5, it also appears that the second‐order dipolar contributions obtained with M06 and M06‐2X are poorly predicted both in sign and magnitude.^[93]^ Finally, the individual components calculated with the hybrid meta‐GGA (TPSSh) are in fair agreement with experiment and compare well with those obtained with hybrid functionals. Overall, the performances of the above functionals are directly connected to their ability to predict adequate isotropic terms. Indeed, there is a strong diversity of results for *A_iso_* (Table 6) and the performances of the functionals may be related to either core‐ (M06, PBE, SCAN, TPSS) or valence‐shell spin polarization (CAM‐B3YP, M06, M06‐2X, SCAN). However, very little spin contamination is observed in general, with GGA and meta‐GGA functionals having the least deviation from the ideal value of 0.75 for a spin *S*=1/2 system. On the contrary, the Minnesota functionals deviate the most from this value, in line with core contributions being described poorly. This performance evaluation becomes obvious when analyzing the data on absolute percent deviations reported in Table 7 and graphically presented in Figure 3.

**Table 5:** Calculated HFCs (total values and individual components, in MHz) for **[Cu(NH_3_)_4_]^2+^** obtained with the aug‐cc‐pVTZ‐Jmod basis set with different functionals.

Functional	Total			Isotropic		Anisotropic					
	A_11_	A_22_	A_33_	A^FC^	A^PC^	A_11_^SD^	A_22_^SD^	A_33_^SD^	A_11_^SO,dip^	A_22_^SO,dip^	A_33_^SO,dip^
B3LYP	−2.0	−2.4	−576.6	−320.2	126.5	248.2	247.7	−495.8	−56.5	−56.4	112.9
B3PW91	−19.2	−19.6	−591.2	−337.9	127.9	248.1	247.6	−495.7	−57.3	−57.2	114.4
CAM‐B3LYP	−56.0	−56.6	−768.3	−338.7	45.0	259.0	258.5	−517.5	−21.4	−21.3	42.8
M06	−91.0	290.2	290.6	−72.9	236.2	−509.8	254.6	255.2	255.6	−127.7	−127.9
M062X	−241.6	−241.9	−518.5	−675.0	341.0	276.0	275.4	−551.4	−183.6	−183.2	366.9
PBE	6.3	6.5	−502.5	−254.8	91.6	210.9	211.2	−422.0	−41.3	−41.4	82.8
PBE0	−23.8	−24.3	−606.4	−359.5	139.1	258.0	257.4	−515.4	−62.4	−62.3	124.8
SCAN	31.4	33.1	−627.6	−254.0	64.6	246.9	248.7	−495.6	−26.2	−26.2	57.4
TPSS	11.4	11.7	−534.2	−254.5	84.1	218.5	218.8	−437.3	−36.7	−36.8	73.5
TPSSh	−5.4	−5.8	−586.3	−298.0	98.8	236.5	236.0	−472.5	−42.7	−42.6	85.3
*Exp*.	*68.8*	*68.8*	*586.5*

**Table 6:** Core and valence‐shell spin contributions (in MHz) for **[Cu(NH_3_)_4_]^2+^** obtained with the aug‐cc‐pVTZ‐Jmod basis set with different functionals.

Functional	**1 s**	**2 s**	**3 s**	Core	Valence	A_iso_	$\left\langle S^{2}\right\rangle$	APD($\left\langle S^{2}\right\rangle$ )
B3LYP	−18.6	−554.4	409.3	−163.7	−30.0	−193.7	0.7524	0.32
B3PW91	−18.0	−567.9	403.0	−182.9	−27.1	−210.0	0.7524	0.32
CAM‐B3LYP	−17.9	−578.0	424.9	−171.0	−122.7	−293.6	0.7526	0.35
M06	−6.4	−217.8	286.6	62.3	100.9	163.3	0.7527	0.36
M062X	−9.1	−1420.6	813.5	−616.2	282.2	−334.0	0.7531	0.41
PBE	−9.7	−439.5	297.1	−15.0	−11.2	−163.2	0.7511	0.15
PBE0	−17.9	−620.7	436.3	−202.3	−15.9	−218.2	0.7527	0.36
SCAN	−9.1	−679.1	622.4	−65.8	−121.9	−187.7	0.7526	0.34
TPSS	−8.8	−409.1	283.2	−65.8	−35.7	−170.4	0.7514	0.18
TPSSh	−12.2	−477.0	335.5	−153.7	−45.5	−199.2	0.7520	0.26
					*Exp*.	*241.4*	*0.7500*

**Table 7:** APDs of *A*_*33*_, *A_iso_* and *ΔA* for calculated HFCs obtained with the aug‐cc‐pVTZ‐Jmod basis set using different functionals with respect to experimental HFCs for **[Cu(NH_3_)_4_]^2+^**.

Functional	A_33_	A_iso_	ΔA	APD(A_33_)	APD(A_iso_)	APD(ΔA)
B3LYP	−576.6	193.7	574.6	1.7	19.8	11.0
B3PW91	−591.2	210.0	572.0	0.8	13.0	10.5
CAM‐B3LYP	−768.3	293.6	712.3	31.0	21.7	37.6
M06	290.6	163.3	381.6	50.5	32.4	26.3
M062X	−518.5	334.0	276.9	11.6	38.4	46.5
PBE	−502.5	163.2	509.0	14.3	32.4	1.7
PBE0	−606.4	218.2	582.6	3.4	9.6	12.5
SCAN	−627.6	187.7	660.7	7.0	22.2	27.6
TPSS	−534.2	170.4	545.9	8.9	29.4	5.4
TPSSh	−586.3	199.2	580.9	0.0	17.5	12.2
*Exp*.	*586.5*	*241.4*	*517.7*

At this first glance, hybrid functionals reproduce very well the magnitude of the largest hyperfine components, *A_33_*, with an average of 1.5 % deviation. The best match is found for the hybrid meta‐GGA TPPSh in the case of [Cu(NH_3_)_4_]^2+^. For this parameter, the meta‐GGA functionals are also competitive with an average of 8 % deviation. As previously mentioned, the other functionals are of poor quality for the prediction of *A_33_*. Interestingly, similar trends are observed for the average value of the HFC, *A_iso_*, where deviations do increase to about 15 % for the (PBE0, B3LYP and B3PW91) group of functionals. Finally, the anisotropic parameter, *ΔA*, is also in good agreement with experiment with an average of 11 % deviation when looking at the hybrid functionals. The performances of the hybrid meta‐GGA, TPSSh, remain correct for both isotropic, *A_iso_*, and anisotropic, *ΔA*, parameters. The worst performing functionals are M06 and M062X while the GGA and meta‐GGA functionals underperform compared to their hybrid counterpart.

**Figure 4:**
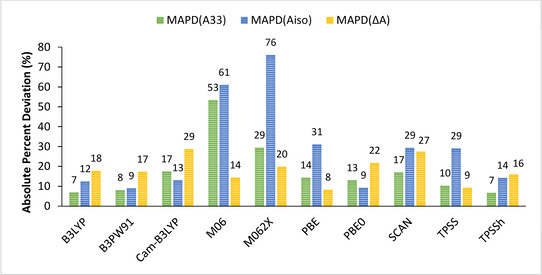
Graphical representation of the mean APDs of *A_33_*, *A_iso_*, and *ΔA* for calculated values of the complete set of complexes obtained with the aug‐cc‐pVTZ‐Jmod basis set and different functionals with respect to experimental results.

For a complete evaluation of the performances of each functional, the calculations were subsequently performed for the complete set of 20 complexes (Tables S27–S45) and the mean of each absolute percent deviation (MAPD) reported in Table 8 and graphically presented in Figure 4 were obtained in the following manner:

$$MAPD\left(A_{33}^{\left(M\right)}\right)=\;\frac{\sum {APD(A}_{33}^{\left(M\right)})\;}{20},\;MAPD\left(A_{iso}^{\left(M\right)}\right)=\frac{\sum {APD(A}_{iso}^{\left(M\right)})\;}{20}\;\;MAPD\left(\Delta A\right)=\;\frac{\sum APD(\Delta A)}{20}$$

**Table 8:** Mean APDs of *A_33_*, *A_iso_* and *ΔA* with respect to experimental results for the complete set of complexes obtained with the aug‐cc‐pVTZ‐Jmod basis set and different functionals.

Functional	MAPD(A_33_)	MAPD(A_iso_)	MAPD(ΔA)
B3LYP	6.9	12.5	17.8
B3PW91	8.1	9.0	17.3
CAM‐B3LYP	17.5	13.0	28.7
M06	53.3	61.0	14.3
M062X	29.4	76.0	19.9
PBE	14.3	31.1	8.2
PBE0	13.0	9.3	21.7
SCAN	17.0	29.3	27.4
TPSS	10.3	29.1	9.2
TPSSh	6.6	14.2	15.9

From these mean absolute percent deviations, we can evaluate the ability of a given functional to predict reliable and accurate hyperfine coupling constants. Using our aug‐cc‐pVTZ‐Jmod basis sets, we obtain the following trend: B3PW91<TPSSh<B3LYP<PBE0<TPSS<PBE<CAM‐B3LYP<SCAN<M062X<M06, with B3PW91 having the lowest deviation from experiment and M06 the largest. Table 8 and Figure 4 both show that B3PW91 is the best performing functional, a conclusion in line with a previous study.^[17]^ This behavior can be extended to the other hybrid (PBE0 and B3LYP) and hybrid meta‐GGA (TPSSh) functionals which feature moderate mean ADPs with respect to experiment. On the other hand, and consistently with previous observations, GGA and meta‐GGA functionals underperform compared to their hybrid counterparts, while the Minnesota functionals cannot be considered applicable for the prediction of HFCs.

To finalize the comparison of DFT methods, we included the double hybrid functional B2PLYP to our study and computed the HFCs for the complete set of complexes. This functional was first evaluated using the mean absolute percent deviations for the Fermi contact term and the three components of the spin dipolar contribution with respect to the ones obtained using the B3PW91 functional and the aug‐cc‐pVTZ‐Jmod basis set. In the specific case of [Cu(NH_3_)_4_]^2+^, Table 9 shows that B2PLYP provide calculated values for the spin‐dipolar terms close to those obtained with B3PW91, but deviates non‐negligibly for the Fermi contact term.

**Table 9:** Hyperfine contributions *A* ^*FC*^ and *A* ^*SD*^ (total values and individual components, in MHz) using the aug‐cc‐pVTZ‐Jmod basis set and the B2PLYP functional and absolute APDs for **[Cu(NH_3_)_4_]^2+^** with respect to B3PW91 values.

	A^FC^	A_11_^SD^	A_22_^SD^	A_33_^SD^	APD(A^FC^)	APD(A_11_^SD^)	APD(A_22_^SD^)	APD(A_33_^SD^)
B2PLYP	−382.7	261.7	261.2	−522.9	13.2	5.5	5.5	5.5
B3PW91	−337.9	248.1	247.6	−495.7

Looking at the entire set of complexes (Tables 9 and S46), the following mean absolute percent deviations were obtained: MAPD(*A* ^*FC*^)=16, MAPD(*A_11_* ^*SD*^)=12, MAPD(*A_22_* ^*SD*^)=8, and MAPD(*A_33_* ^*SD*^)=10. On average, the value that deviates the most is for the Fermi contact term while the predicted spin‐dipolar terms remain within an acceptable range of deviation, *i. e*. 10 % on average. This initial comparison between B2PLYP with B3PW91 only gives an estimate on the ability of this double hybrid functional to predict HFCs. However, due to the lack of the spin‐orbit contribution, which is not implemented for the B2PLYP method, the total hyperfine coupling constants cannot be directly calculated. To overcome this, we simply added the individual spin‐orbit terms, *A* ^*SO*^, calculated with B3PW91 to the remaining B2PLYP components and obtain fully comparable hyperfine tensors for the entire set of complexes.

While it seems promising for the case of [Cu(NH_3_)_4_]^2+^, the HFCs obtained with B2PLYP using the SOC contributions from B3PW91 are well above an acceptable error range over the complete set of complexes (Table S47). Table 10 finally reports the mean absolute percent deviations for the 20 complexes and we observe that the errors are evenly distributed between the studied parameters *A_33_*, *A_iso_* and *ΔA* making B2PLYP an unfit DFT method to predict Cu hyperfine coupling constants.

**Table 10:** Mean APDs of *A_33_*, *A_iso_* and *ΔA* with respect to experimental results for the complete complex set obtained with B2PLYP using B3PW91 *A* ^*SO*^ values and the aug‐cc‐pVTZ‐Jmod basis set.

Functional	MAPD(A_33_)	MAPD(A_iso_)	MAPD(ΔA)
B2PLYP	19.4	19.5	24.7
B3PW91	8.1	9.0	17.3

### 2.4 Performance of Wave Function Methods

Most of the correlated wave function calculations required an auxiliary basis set for the resolution of the identity approximations that are used either optionally or by default, as for example in the OO methods and in DLPNO‐CCSD. Standard main basis sets often have custom‐made auxiliary bases for correlation fitting, denoted as /C. This is the case for the def2 family and the correlation consistent (cc‐pV*n*Z) series of basis sets. However, neither the standard basis sets identified in the benchmark DFT calculations as potential candidates for the calculation of copper hyperfine coupling constants, nor our final choice defined above (aug‐cc‐pVTZ‐Jmod) have dedicated auxiliary basis sets. It is noted that for the aug‐cc‐pVTZ‐J basis set it appears that the standard aug‐cc‐pVTZ/C is sufficient, both in non‐relativistic and DKH2 scalar relativistic calculations. In comparison, the aug‐cc‐pwCVTZ/C and the larger aug‐cc‐pVQZ/C auxiliary basis perform less well, demonstrating the low tolerances involved in adapting a correlation fitting basis set to a specific size and type of main basis. It is also noted that the def2/C basis sets are inadequate when either the aug‐cc‐pVTZ‐J or the smaller CP(PPP) were used as main basis sets for Cu. Modifications of standard basis sets to make them more appropriate for aug‐cc‐pVTZ‐Jmod did not show a systematic trend, therefore we opted instead for the automatic generation procedure encoded in ORCA,^[65]^ which led to a [38s37p37d35f35g7h5i] auxiliary that reproduced exactly the non‐RI energies and properties.

**Figure 5:**
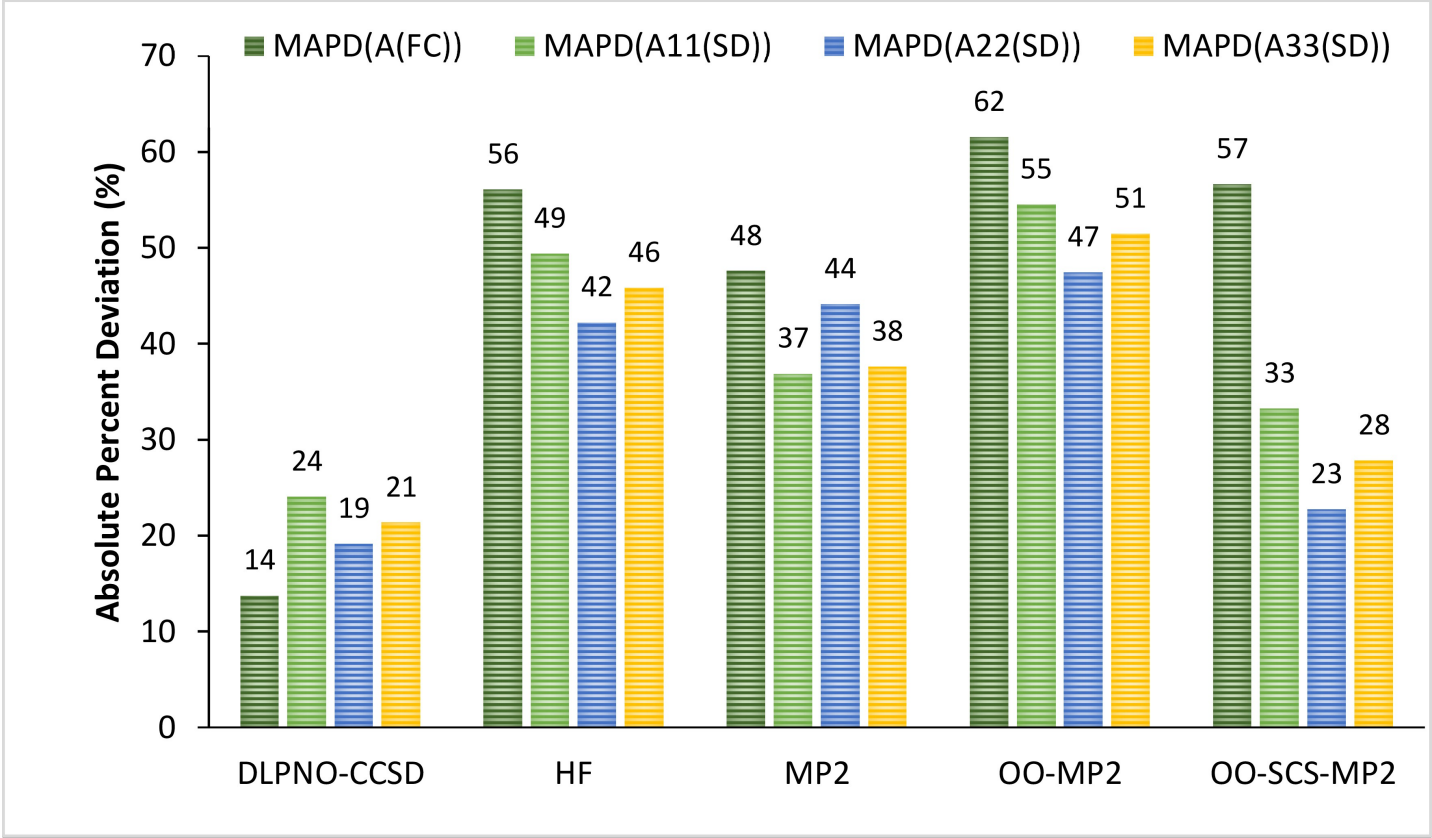
Graphical representation of the mean APDs of *A* ^*FC*^ and *A* ^*SD*^ for calculated values of the complete set of complexes using the aug‐cc‐pVTZ‐Jmod basis set and different wave function methods with respect to B3PW91 values.

The wave function methods studied here for the evaluation of hyperfine coupling constants can only provide the *A* ^*FC*^ and the *A* ^*SD*^ terms. Given the impossibility to calculate the complete hyperfine tensor with these wave‐function based methods, we decided to compare their performances to the best performing DFT functional with respect to experimental values. A first evaluation was therefore carried out with respect to the corresponding terms obtained with B3PW91. The resulting data are presented in Table 11 and Figure 5 for the mean absolute percent deviations and detailed contributions are reported in Supplementary Information (Table S48).

**Table 11:** Hyperfine contributions *A* ^*FC*^ and *A* ^*SD*^ (individual components, in MHz) and APDs for the complete set of complexes using the aug‐cc‐pVTZ‐Jmod basis set and different wave function methods, with respect to B3PW91values.

Wave function method	MAPD(A^FC^)	MAPD(A_11_^SD^)	MAPD(A_22_^SD^)	MAPD(A_33_^SD^)
DLPNO‐CCSD	13.7	24.1	19.1	21.4
HF	56.1	49.4	42.2	45.8
MP2	47.6	36.8	44.1	37.6
OO‐MP2	61.5	54.5	47.4	51.4
OO‐SCS‐MP2	56.7	33.2	22.7	27.8

The Fermi contact term appears to be the most sensitive parameter for HF, MP2, OO‐MP2 and OO‐SCS‐MP2 methods. With the exception of OO‐SCS‐MP2, the calculated spin‐dipolar contributions also deviate quite significantly from the B3PW91 reference values. On the other hand, the opposite trend is observed for DLPNO‐CCSD which is the best performing wave function based method since it displays the smallest mean deviations.

**Figure 6:**
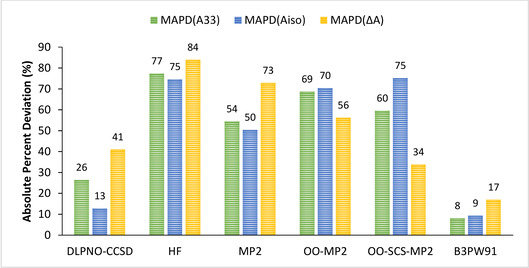
Graphical representation of the mean APDs of *A_33_*, *A_iso_* and *ΔA* for calculated values of the complete set of complexes obtained with the aug‐cc‐pVTZ‐Jmod basis set and different wave function methods, using B3PW91 *A* ^*SO*^ values, with respect to experimental results.

A second evaluation was conducted with respect to experimental results. In this case, the *A* ^*SO*^ obtained from B3PW91 is applied to each wave function method to yield complete hyperfine coupling constants. Table 12 and Figure 6 finally reports the mean absolute percent deviations for the calculated HFCs of the 20 complexes.

**Table 12:** Mean APDs of *A_33_*, *A_iso_* and *ΔA* with respect to experimental results for the complete complex set obtained with different wave function methods using B3PW91 *A* ^*SO*^ values and the aug‐cc‐pVTZ‐Jmod basis set.

Wave function method	MAPD(A_33_)	MAPD(A_iso_)	MAPD(ΔA)
DLPNO‐CCSD	26.4	12.8	41.0
HF	77.4	74.6	83.9
MP2	54.4	50.4	72.9
OO‐MP2	68.7	70.4	56.2
OO‐SCS‐MP2	59.6	75.1	33.8
B3PW91	8.1	9.0	17.3

Once the SOC terms have been added and the total components evaluated against experimental values, a general trend in absolute percent deviation can be extracted: B3PW91<DLPNO‐CCSD<OO‐SCS‐MP2<MP2<OO‐MP2<HF, with B3PW91 having the lowest deviation from experiment and HF the largest. B3PW91 continues to perform much better as a DFT method compared to any other *ab initio* methods.

A closer look at these data also show that DLPNO‐CCSD offers the best results with a reasonably low deviation for the prediction of the isotropic hyperfine contribution. The OO‐SCS‐MP2 is the second‐best performing method due to its low *ΔA* deviation. On the contrary, the MAPD deviation of both the *A_33_* and *A_iso_* terms is quite high and of similar magnitude than the ones obtained with OO‐MP2. The opposite trend is found for the MP2 case: the *ΔA* deviation is greater than the one obtained for *A_33_* and *A_iso_*. The simple HF calculation is consistent throughout the terms but highly inaccurate.

Overall, none of the wave‐function based methods is able to compete with B3PW91 for the prediction of accurate HFCs in an average sense. The only promising approach among the methods examined here is DLPNO‐CCSD, but this still lags behind DFT. It will be interesting to see whether future developments, for example by incorporating the effects of triples excitations or using improved densities, can offer better control of errors and thereby improve the performance of this approach.

## 3 Conclusions

In this work we evaluated the performance of density functional theory and modern wave function based methods for the prediction of Cu hyperfine coupling constants using a large set of 20 complexes with experimentally known structures and resolved hyperfine coupling tensors. We defined a computationally optimal basis set for Cu, the aug‐cc‐pVTZ‐Jmod basis set, a modified version of aug‐cc‐pVTZ‐J with decontracted *s* functions and removal of innermost *s* primitives, as a robust, flexible and generally applicable basis set that can be used with all quantum chemical method tested in the present study. With this basis set we carried out an unbiased evaluation of a series of DFT methods representing all families of functionals, and of wave function based methods. Our results indicated that the hybrid functional B3PW91 performs best as it features the lowest mean absolute deviation of the total HFC with respect to experimental data. It was also shown that, to a lesser extent, conventional hybrid and hybrid meta‐GGA functionals such as TPSSh, B3LYP and PBE0 are also able to provide accurate and reliable hyperfine tensors. On the contrary, more modern functionals, such as the range‐separated CAM‐B3LYP or those of the Minnesota group, yield poor estimations of the total HFCs. Consistently with previous reports, net underestimations were observed with GGA while meta‐GGA functionals underperform compared to their hybrid counterparts. The double hybrid functional B2PLYP was also included in this study and when evaluated against the reference functional B3PW91, the resulting mean error showed that B2PLYP cannot deliver a reliable prediction of Cu HFCs. A similar conclusion is reached when investigating the performance of wave function based methods. Currently these approaches only calculate the Fermi contact and spin‐dipolar terms. To overcome this limitation, the SOC contribution from B3PW91 was added to generate complete hyperfine coupling constants. Within this approximation, the DLPNO‐CCSD method provided the best results, but does not compete with hybrid and hybrid meta‐GGA functionals. In conclusion, and despite its shortcomings, DFT remains the method of choice for the calculation of Cu(II) hyperfine coupling constants.

## Conflict of interest

The authors declare no conflict of interest.

## Supporting information

As a service to our authors and readers, this journal provides supporting information supplied by the authors. Such materials are peer reviewed and may be re‐organized for online delivery, but are not copy‐edited or typeset. Technical support issues arising from supporting information (other than missing files) should be addressed to the authors.

Supplementary

## References

[1] E. I. Solomon , U. M. Sundaram , T. E. Machonkin , Chem. Rev. 1996, 96, 2563–2606. 11848837 10.1021/cr950046o

[2] R. L. Peterson , S. Kim , K. D. Karlin , in Comprehensive Inorganic Chemistry II (Second Edition) (Eds.: J. Reedijk, K. Poeppelmeier), Elsevier, Amsterdam, 2013, pp. 149–177.

[3] R. Trammell , K. Rajabimoghadam , I. Garcia-Bosch , Chem. Rev. 2019, 119, 2954–3031. 30698952 10.1021/acs.chemrev.8b00368 PMC6571019

[4] E. I. Solomon , D. E. Heppner , E. M. Johnston , J. W. Ginsbach , J. Cirera , M. Qayyum , M. T. Kieber-Emmons , C. H. Kjaergaard , R. G. Hadt , L. Tian , Chem. Rev. 2014, 114, 3659–3853. 24588098 10.1021/cr400327t PMC4040215

[5] G. Tabbì , A. Giuffrida , R. P. Bonomo , J. Inorg. Biochem. 2013, 128, 137–145. 23988848 10.1016/j.jinorgbio.2013.07.035

[6] E. Garribba , G. Micera , J. Chem. Educ. 2006, 83, 1229.

[7] J. Peisach , W. E. Blumberg , Arch. Biochem. Biophys. 1974, 165, 691–708. 4374138 10.1016/0003-9861(74)90298-7

[8] U. Sakaguchi , A. W. Addison , J. Chem. Soc., Dalton Trans. 1979, 600–608.

[9] P. Bertrand , Electron Paramagnetic Resonance Spectroscopy: Fundamentals, Springer International Publishing, 2020, p 420 .

[10] P. Bertrand , Electron Paramagnetic Resonance Spectroscopy: Applications, Springer International Publishing, 2020.

[11] M. Kaupp , M. Bühl , V. G. Malkin , Calculation of NMR and EPR Parameters: Theory and Applications, Wiley-VCH, Weinheim, Germany, 2004, p. 603.

[12] F. Neese , J. Chem. Phys. 2003, 118, 3939–3948.

[13] F. Neese , Coord. Chem. Rev. 2009, 253, 526–563.

[14] M. Munzarová , M. Kaupp , J. Phys. Chem. A 1999, 103, 9966–9983.

[15] E. Malkin , I. Malkin , O. L. Malkina , V. G. Malkin , M. Kaupp , Phys. Chem. Chem. Phys. 2006, 8, 4079–4085. 17028696 10.1039/b607044b

[16] S. Kossmann , B. Kirchner , F. Neese , Mol. Phys. 2007, 105, 2049–2071.

[17] G. Sciortino , G. Lubinu , J.-D. Maréchal , E. Garribba , Magnetochemistry 2018, 4, 55.

[18] D. A. Pantazis , Magnetochemistry 2019, 5, 69.

[19] C. Remenyi , R. Reviakine , A. V. Arbuznikov , J. Vaara , M. Kaupp , J. Phys. Chem. A 2004, 108, 5026–5033.

[20] W. M. Ames , S. C. Larsen , Phys. Chem. Chem. Phys. 2009, 11, 8266–8274. 19756283 10.1039/b905750a

[21] S. Kossmann , F. Neese , J. Phys. Chem. A 2010, 114, 11768–11781. 20931951 10.1021/jp105647c

[22] B. Sandhoefer , S. Kossmann , F. Neese , J. Chem. Phys. 2013, 138, 104102–104115. 23514460 10.1063/1.4792362

[23] M. Saitow , F. Neese , J. Chem. Phys. 2018, 149, 034104. 30037259 10.1063/1.5027114

[24] T. Shiozaki , T. Yanai , J. Chem. Theory Comput. 2016, 12, 4347–4351. 27479148 10.1021/acs.jctc.6b00646

[25] W. M. Ames , S. C. Larsen , J. Phys. Chem. A 2010, 114, 589–594. 20000556 10.1021/jp907878h

[26] S. Sinnecker , F. Neese , J. Comput. Chem. 2006, 27, 1463–1475. 16807973 10.1002/jcc.20426

[27] C. Remenyi , R. Reviakine , M. Kaupp , J. Phys. Chem. B 2007, 111, 8290–8304. 17592871 10.1021/jp071745v

[28] C. Remenyi , R. Reviakine , M. Kaupp , J. Phys. Chem. A 2006, 110, 4021–4033. 16539425 10.1021/jp057594i

[29] F. Neese , F. Wennmohs , U. Becker , C. Riplinger , J. Chem. Phys. 2020, 152, 224108. 32534543 10.1063/5.0004608

[30] A. D. Becke , Phys. Rev. A 1988, 38, 3098–3100. 10.1103/physreva.38.3098 9900728

[31] J. P. Perdew , Phys. Rev. B 1986, 33, 8822–8824. 10.1103/physrevb.33.8822 9938299

[32] F. Weigend , R. Ahlrichs , Phys. Chem. Chem. Phys. 2005, 7, 3297–3305. 16240044 10.1039/b508541a

[33] F. Weigend , Phys. Chem. Chem. Phys. 2006, 8, 1057–1065. 16633586 10.1039/b515623h

[34] J. P. Perdew , K. Burke , M. Ernzerhof , Phys. Rev. Lett. 1996, 77, 3865–3868. 10062328 10.1103/PhysRevLett.77.3865

[35] J. Tao , J. P. Perdew , V. N. Staroverov , G. E. Scuseria , Phys. Rev. Lett. 2003, 91, 146401. 14611541 10.1103/PhysRevLett.91.146401

[36] J. Sun , A. Ruzsinszky , J. P. Perdew , Phys. Rev. Lett. 2015, 115, 036402. 26230809 10.1103/PhysRevLett.115.036402

[37] A. D. Becke , J. Chem. Phys. 1993, 98, 5648–5652.

[38] C. Lee , W. Yang , R. G. Parr , Phys. Rev. B 1988, 37, 785–789. 10.1103/physrevb.37.785 9944570

[39] T. Yanai , D. P. Tew , N. C. Handy , Chem. Phys. Lett. 2004, 393, 51–57.

[40] J. P. Perdew , Y. Wang , Phys. Rev. B 1992, 45, 13244–13249. 10.1103/physrevb.45.13244 10001404

[41] C. Adamo , V. Barone , J. Chem. Phys. 1999, 110, 6158–6170.

[42] V. N. Staroverov , G. E. Scuseria , J. Tao , J. P. Perdew , J. Chem. Phys. 2003, 119, 12129–12137.

[43] Y. Zhao , D. G. Truhlar , Theor. Chem. Acc. 2008, 120, 215–241.

[44] S. Grimme , J. Chem. Phys. 2006, 124, 034108. 16438568 10.1063/1.2148954

[45] F. Neese , J. Chem. Phys. 2005, 122, 034107. 10.1063/1.1829047 15740192

[46] B. A. Hess , C. M. Marian , U. Wahlgren , O. Gropen , Chem. Phys. Lett. 1996, 251, 365–371.

[47] M. Douglas , N. M. Kroll , Ann. Phys. 1974, 82, 89–155.

[48] B. A. Hess , Phys. Rev. A 1985, 32, 756–763. 10.1103/physreva.32.756 9896123

[49] B. A. Hess , Phys. Rev. A 1986, 33, 3742–3748. 10.1103/physreva.33.3742 9897114

[50] G. Jansen , B. A. Hess , Phys. Rev. A 1989, 39, 6016–6017. 10.1103/physreva.39.6016 9901188

[51] A. Wolf , M. Reiher , B. A. Hess , J. Chem. Phys. 2002, 117, 9215–9226.

[52] M. Reiher , Theor. Chem. Acc. 2006, 116, 241–252.

[53] T. Nakajima , K. Hirao , Chem. Rev. 2012, 112, 385–402. 21678899 10.1021/cr200040s

[54] E. van Lenthe , E. J. Baerends , J. G. Snijders , J. Chem. Phys. 1993, 99, 4597–4610.

[55] E. van Lenthe , E. J. Baerends , J. G. Snijders , J. Chem. Phys. 1994, 101, 9783–9792.

[56] E. van Lenthe , J. G. Snijders , E. J. Baerends , J. Chem. Phys. 1996, 105, 6505–6516.

[57] D. A. Pantazis , F. Neese , WIREs Comput. Mol. Sci. 2014, 4, 363–374.

[58] L. Visscher , K. G. Dyall , Atom. Data Nucl. Data Tabl. 1997, 67, 207–224.

[59] R. C. Lochan , M. Head-Gordon , J. Chem. Phys. 2007, 126, 164101. 17477583 10.1063/1.2718952

[60] F. Neese , T. Schwabe , S. Kossmann , B. Schirmer , S. Grimme , J. Chem. Theory Comput. 2009, 5, 3060–3073. 26609985 10.1021/ct9003299

[61] C. Riplinger , F. Neese , J. Chem. Phys. 2013, 138, 034106. 23343267 10.1063/1.4773581

[62] M. Saitow , U. Becker , C. Riplinger , E. F. Valeev , F. Neese , J. Chem. Phys. 2017, 146, 164105. 28456208 10.1063/1.4981521

[63] A. Hellweg , C. Hättig , S. Höfener , W. Klopper , Theor. Chem. Acc. 2007, 117, 587–597.

[64] E. D. Hedegård , J. Kongsted , S. P. A. Sauer , J. Chem. Theory Comput. 2011, 7, 4077–4087. 26598353 10.1021/ct200587k

[65] G. L. Stoychev , A. A. Auer , F. Neese , J. Chem. Theory Comput. 2017, 13, 554–562. 28005364 10.1021/acs.jctc.6b01041

[66] H. J. Scholl , J. Hüttermann , J. Phys. Chem. 1992, 96, 9684–9691.

[67] Y. Suzuki , S. Fujii , T. Tominaga , T. Yoshimoto , T. Yoshimura , H. Kamada , Biochim. Biophys. Acta 1997, 1335, 242–245. 9202186 10.1016/s0304-4165(97)00027-5

[68] N. Ritterskamp , K. Sharples , E. Richards , A. Folli , M. Chiesa , J. A. Platts , D. M. Murphy , Inorg. Chem. 2017, 56, 11862–11875. 28933856 10.1021/acs.inorgchem.7b01874

[69] E. Carter , E. L. Hazeland , D. M. Murphy , B. D. Ward , Dalton Trans. 2013, 42, 15088–15096. 24000097 10.1039/c3dt51694f

[70] K. Bader , D. Dengler , S. Lenz , B. Endeward , S.-D. Jiang , P. Neugebauer , J. van Slageren , Nat. Commun. 2014, 5, 5304. 25328006 10.1038/ncomms6304

[71] W. E. Antholine , R. Basosi , J. S. Hyde , S. Lyman , D. H. Petering , Inorg. Chem. 1984, 23, 3543–3548.

[72] I. Kuźniarska-Biernacka , K. Kurzak , B. Kurzak , J. Jezierska , J. Solution Chem. 2003, 32, 719–741.

[73] J. García-Tojal , A. García-Orad , J. L. Serra , J. L. Pizarro , L. Lezama , M. I. Arriortua , T. Rojo , J. Inorg. Biochem. 1999, 75, 45–54. 10402676 10.1016/S0162-0134(99)00031-8

[74] N. Singh , J. Niklas , O. Poluektov , K. M. Van Heuvelen , A. Mukherjee , Inorg. Chim. Acta 2017, 455, 221–230.

[75] S. Sarkar , S. Sen , S. Dey , E. Zangrando , P. Chattopadhyay , Polyhedron 2010, 29, 3157–3163.

[76] K. M. Ananth , M. Kanthimathi , B. U. Nair , Transition Met. Chem. 2001, 26, 333–338.

[77] P. Comba , T. W. Hambley , G. A. Lawrance , L. L. Martin , P. Renold , K. Váarnagy , J. Chem. Soc., Dalton Trans. 1991, 277–283.

[78] M. M. Bhadbhade , D. Srinivas , Inorg. Chem. 1993, 32, 5458–5466.

[79] B. Balaiah , B. A. Sastry , M. N. Chary , G. Ponticelli , M. Massacesi , J. Mol. Struct. 1982, 78, 289–297.

[80] R. S. Glass , L. K. Steffen , D. D. Swanson , G. S. Wilson , R. de Gelder , R. A. G. de Graaff , J. Reedijk , Inorg. Chim. Acta 1993, 207, 241–252.

[81] F. Neese , Inorg. Chim. Acta 2002, 337 C, 181–192.

[82] E. D. Hedegard , J. Kongsted , S. P. A. Sauer , Phys. Chem. Chem. Phys. 2012, 14, 10669–10676. 22785432 10.1039/c2cp40969k

[83] A. Schäfer , C. Huber , R. Ahlrichs , J. Chem. Phys. 1994, 100, 5829–5835.

[84] H. S. Shafaat , J. J. Griese , D. A. Pantazis , K. Roos , C. S. Andersson , A. Popović-Bijelić , A. Gräslund , P. E. M. Siegbahn , F. Neese , W. Lubitz , M. Högbom , N. Cox , J. Am. Chem. Soc. 2014, 136, 13399–13409. 25153930 10.1021/ja507435t

[85] D. A. Pantazis , X. Y. Chen , C. R. Landis , F. Neese , J. Chem. Theory Comput. 2008, 4, 908–919. 26621232 10.1021/ct800047t

[86] D. A. Pantazis , F. Neese , J. Chem. Theory Comput. 2009, 5, 2229–2238. 26616609 10.1021/ct900090f

[87] D. A. Pantazis , F. Neese , J. Chem. Theory Comput. 2011, 7, 677–684.

[88] D. A. Pantazis , F. Neese , Theor. Chem. Acc. 2012, 131, 1292.

[89] D. Aravena , F. Neese , D. A. Pantazis , J. Chem. Theory Comput. 2016, 12, 1148–1156. 26839966 10.1021/acs.jctc.5b01048

[90] J. D. Rolfes , F. Neese , D. A. Pantazis , J. Comput. Chem. 2020, 41, 1842–1849. 32484577 10.1002/jcc.26355

[91] A. C. Saladino , S. C. Larsen , J. Phys. Chem. A 2003, 107, 5583–5587.

[92] C. J. Schattenberg , T. M. Maier , M. Kaupp , J. Chem. Theory Comput. 2018, 14, 5653–5672. 30299950 10.1021/acs.jctc.8b00597

[93] A. Wodyński , M. Kaupp , J. Chem. Theory Comput. 2020, 16, 314–325. 31834796 10.1021/acs.jctc.9b00911

