# Supplementary material for: Comparison of Density Functional and Correlated Wave Function Methods for the Prediction of Cu(II) Hyperfine Coupling Constants
Source: Chemphyschem. 2020 Nov 17;21(24):2667–79. doi: 10.1002/cphc.202000649 (PMC7756273; doi:10.1002/cphc.202000649)
Supplement: Supplementary file 1 — Supplementary [file CPHC-21-2667-s001.pdf]

ChemPhysChem

Supporting Information

Comparison of Density Functional and Correlated Wave Function Methods for the Prediction of Cu(II) Hyperfine Coupling Constants

Rogelio J. Gómez-Piñeiro, Dimitrios A. Pantazis,* and Maylis Orio*

SUPPORTING INFORMATION

Table of Contents

Table S1. Evaluation of the Coulomb term of the spin-orbit coupling contribution for the $[Cu(NH_3)_4]^{2+}$ complex using PBE0 and different basis sets.

Tables S2-S3. Solvation effects on $[Cu(NH_3)_4]^{2+}$ complex using CPCM, B3PW91 and the modified version of aug-cc-pVTZ-J.

Tables S4-S7 and Figures S1-2. Optimization of basis set for $[Cu(NH_3)_4]^{2+}$ complex using B3PW91 and TPSSh.

Tables S8-S13 and Figures S3-S5. Optimization of basis set for $[Cu(dtc)_2]$ complex using PBE0, B3PW91, and TPSSh.

Tables S14-S19 and Figures S6-S9. Optimization of basis set for $[Cu(acac)_2]$ complex using PBE0, B3PW91, and TPSSh.

Tables S20-S21. DKH effects on the hyperfine tensors of the $[Cu(NH_3)_4]^{2+}$ complex using PBE0 and B3PW91.

Tables S22-S23. DKH with Finite Nucleus approximation effects on the hyperfine tensors of the $[Cu(NH_3)_4]^{2+}$ complex using PBE0 and B3PW91.

Tables S24-S26. ZORA effects on the hyperfine tensors of the $[Cu(NH_3)_4]^{2+}$ complex using PBE0 and B3PW91.

Tables S27-S45. Optimization of functionals with complete set of complexes using the modified version of the aug-cc-pVTZ-J basis set.

Tables S46-S47. Performance evaluation of B2PLYP with respect to B3PW91 and experimental results for the complete set of complexes using the modified version of the aug-cc-pVTZ-J.

Table S48. Performance evaluation of wave-function methods with respect to B3PW91 and experimental results for the complete set of complexes using the modified version of the aug-cc-pVTZ-J.

Table S1. Calculated HFCs (individual components and detailed contributions, in MHz) of **$[Cu(NH_3)_4]^{2+}$** obtained with the PBE0 functional with selected basis sets varying the computational method for obtaining the Coulomb term of the SOC operator.

Semi-Numerical	A_{11}	A_{22}	A_{33}	A^{FC}	A^{PC}	A_{11}^{SD}	A_{22}^{SD}	A_{33}^{SD}	$A_{11}^{SO,dip}$	$A_{22}^{SO,dip}$	$A_{33}^{SO,dip}$	***Run Time***
aug-cc-pVTZ-Junc	-26.3	-26.8	-610.3	-360.8	139.7	257.3	256.7	-514.0	-62.4	-62.3	124.7	00:13:37
aug-cc-pVTZ-J	-24.8	-25.2	-607.4	-359.9	140.8	257.2	256.7	-513.9	-62.9	-62.8	125.6	00:15:16
CP(PPP)	-33.2	-33.6	-602.4	-357.8	134.7	249.6	249.1	-498.8	-59.7	-59.6	119.5	00:06:12
aug-cc-pVTZ-Jmod	-23.8	-24.3	-606.4	-359.5	140.8	258.0	257.4	-515.4	-62.9	-62.7	125.7	00:14:38
Semi-Numerical with RI	A_{11}	A_{22}	A_{33}	A^{FC}	A^{PC}	A_{11}^{SD}	A_{22}^{SD}	A_{33}^{SD}	$A_{11}^{SO,dip}$	$A_{22}^{SO,dip}$	$A_{33}^{SO,dip}$	***Run Time***
aug-cc-pVTZ-Junc	-100.5	-101.1	-870.0	-360.8	3.6	257.3	256.7	-514.0	-0.6	-0.6	1.1	00:40:29
aug-cc-pVTZ-J	-99.6	-100.2	-869.1	-359.9	3.6	257.2	256.7	-513.9	-0.6	-0.6	1.2	00:13:48
CP(PPP)	-105.4	-105.9	-854.6	-357.8	2.5	249.6	249.1	-498.8	0.3	0.3	-0.5	00:11:00
aug-cc-pVTZ-Jmod	-98.4	-99.0	-870.1	-359.5	3.6	258.0	257.4	-515.4	-0.5	-0.5	1.2	00:16:41
Exact	A_{11}	A_{22}	A_{33}	A^{FC}	A^{PC}	A_{11}^{SD}	A_{22}^{SD}	A_{33}^{SD}	$A_{11}^{SO,dip}$	$A_{22}^{SO,dip}$	$A_{33}^{SO,dip}$	***Run Time***
aug-cc-pVTZ-Junc	-26.3	-26.7	-610.2	-360.8	140.8	257.3	256.7	-514.0	-62.9	-62.7	125.7	03:21:27
aug-cc-pVTZ-J	-24.7	-25.2	-607.3	-359.9	140.9	257.2	256.7	-513.9	-62.9	-62.8	125.7	06:07:38
CP(PPP)	-33.2	-33.6	-602.3	-357.8	134.8	249.6	249.1	-498.8	-59.8	-59.7	119.5	00:22:02
aug-cc-pVTZ-Jmod	-24.8	-25.2	-610.9	-359.5	139.2	258.0	257.4	-515.4	-62.5	-62.4	124.8	05:03:36

Table S2. Calculated HFCs (individual components and detailed contributions, in MHz) of [Cu(NH3)4]2+ obtained with the B3PW91 and the aug-cc-pVTZ-J-mod basis set, using the CPCM model and different solvents.

Solvent	***Dielectric Constant***	***Refractive Index***	A_{11}	A_{22}	A_{33}	A^{FC}	A^{PC}	A_{11}^{SD}	A_{22}^{SD}	A_{33}^{SD}	$A_{11}^{SO,dip}$	$A_{22}^{SO,dip}$	$A_{33}^{SO,dip}$
THF	7.25	1.407	-13.9	-14.3	-588.4	-335.5	129.9	249.6	249.0	-498.6	-57.9	-57.8	115.8
CH_2Cl_2	9.08	1.424	-13.7	-14.1	-588.2	-335.4	130.0	249.6	249.1	-498.7	-58.0	-57.9	115.8
Methanol	32.63	1.329	-13.1	-13.5	-587.9	-335.1	130.3	249.8	249.3	-499.1	-58.0	-57.9	116.0
Acetonitrile	36.60	1.344	-13.0	-13.5	-587.9	-335.1	130.3	249.8	249.3	-499.1	-58.0	-57.9	116.0
DMSO	47.20	1.479	-13.0	-13.4	-587.9	-335.0	130.3	249.8	249.3	-499.1	-58.0	-57.9	116.0
Water	80.40	1.330	-12.9	-13.3	-587.8	-335.0	130.3	249.8	249.3	-499.1	-58.1	-57.9	116.0

Table S3. APDs of A_{33}, A_{iso} and ΔA of **$[Cu(NH_3)_4]^{2+}$** for calculated hyperfine coupling constants obtained with the B3PW91 functional and the aug-cc-pVTZ-J-mod basis set, the CPCM model and different solvents, and compared to gas phase values.

Solvent	***Dielectric Constant***	***Refractive Index***	A_{33}	A_{iso}	***ΔA***	***APD(A_{33})***	***APD(A_{iso})***	***APD(ΔA)***
THF	7.25	1.407	-588.4	205.5	574.5	0	2	0
CH_2Cl_2	9.08	1.424	-588.2	205.3	574.6	1	2	0
Methanol	32.63	1.329	-587.9	204.8	574.9	1	2	1
Acetonitrile	36.60	1.344	-587.9	204.8	574.9	1	2	1
DMSO	47.20	1.479	-587.9	204.8	574.9	1	2	1
Water	80.40	1.330	-587.8	204.7	574.9	1	3	1
g-phase			-591.2	210.0	572.0			

Table S4. Calculated HFCs (individual components and detailed contributions, in MHz) of $\mathbf{[Cu(NH_3)_4]^{2+}}$ obtained with the B3PW91 functional with different basis sets.

Basis set	A_{11}	A_{22}	A_{33}	A^{FC}	A^{PC}	A_{11}^{SD}	A_{22}^{SD}	A_{33}^{SD}	$A_{11}^{SO,dip}$	$A_{22}^{SO,dip}$	$A_{33}^{SO,dip}$
aug-cc-pVTZ-Junc	-20.7	-21.1	-590.6	-339.2	128.3	247.4	246.9	-494.3	-57.2	-57.2	114.5
aug-cc-pVTZ-J	-19.0	-19.4	-587.5	-338.1	129.5	247.4	246.9	-494.2	-57.8	-57.7	115.3
CP(PPP)	-28.2	-28.6	-583.3	-336.8	123.4	239.8	239.4	-479.3	-54.7	-54.6	109.3
aug-cc-pVTZ	-272.7	325.4	325.8	-5.7	131.8	-515.3	257.4	257.9	116.4	-58.1	-58.2
aug-cc-pwCVTZ	213.0	213.4	-356.1	-104.5	127.9	246.7	247.2	-493.9	-57.1	-57.2	114.4
cc-pwCVTZ	213.9	214.3	-354.7	-103.2	127.7	246.4	246.8	-493.2	-56.9	-57.0	114.1
def2-TZVP	-246.3	332.5	332.9	9.2	130.5	-499.5	249.5	250.0	113.5	-56.7	-56.8
def2-TZVP-uncS	-1.0	-1.3	-580.4	-324.9	130.7	250.1	249.7	-499.8	-56.9	-56.8	113.7
aug-cc-pVTZ-Jmod	-19.2	-19.6	-591.2	-337.9	127.9	248.1	247.6	-495.7	-57.3	-57.2	114.4
Exp.	*68.8*	*68.8*	*586.5*								

Table S5. APDs of A_{33}, A_{iso} and ΔA of $\mathbf{[Cu(NH_3)_4]^{2+}}$ for calculated hyperfine coupling constants obtained with the B3PW91 functional using different basis sets and compared to experimental values.

Basis set	A_{33}	A_{iso}	ΔA	*APD*(A_{33})	*APD*(A_{iso})	*APD*(ΔA)
aug-cc-pVTZ-Junc	-590.6	210.8	569.9	1	13	10
aug-cc-pVTZ-J	-587.5	208.6	568.5	0	14	10
CP(PPP)	-583.3	213.4	555.1	1	12	7
aug-cc-pVTZ	325.8	126.2	598.5	44	48	16
aug-cc-pwCVTZ	-356.1	23.4	569.5	39	90	10
cc-pwCVTZ	-354.7	24.5	569.0	40	90	10
def2-TZVP	332.9	139.7	579.2	43	42	12
def2-TZVP-uncS	-580.4	194.2	579.4	1	20	12
aug-cc-pVTZ-Jmod	-591.2	210.0	572.0	1	13	10
Exp.	*586.5*	*241.4*	*517.7*			

Figure S6. Graphical representation of APDs of A_{33}, A_{iso} and ΔA of $\mathbf{[Cu(NH_3)_4]^{2+}}$ for calculated hyperfine coupling constants obtained with the B3PW91 functional and different basis.

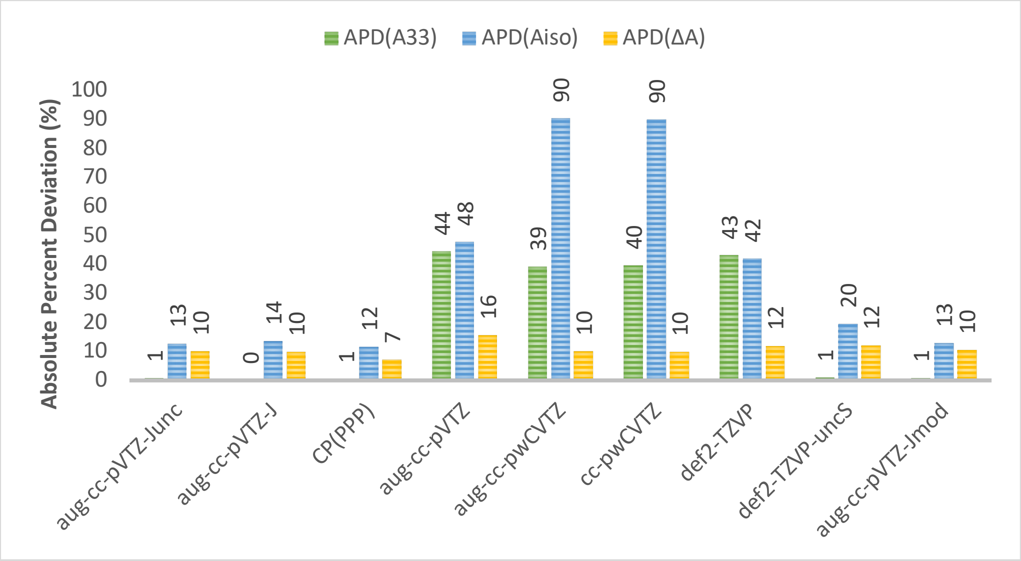

Table S6. Calculated HFCs (individual components and detailed contributions, in MHz) of **$[Cu(NH_3)_4]^{2+}$** obtained with the TPSSh functional with different basis sets.

Basis set	A_{11}	A_{22}	A_{33}	A^{FC}	A^{PC}	A_{11}^{SD}	A_{22}^{SD}	A_{33}^{SD}	$A_{11}^{SO,dip}$	$A_{22}^{SO,dip}$	$A_{33}^{SO,dip}$
aug-cc-pVTZ-Junc	-2.4	-2.8	-580.0	-294.7	99.6	235.5	235.1	-470.6	56.7	56.8	185.4
aug-cc-pVTZ-J	-13.7	-14.1	-589.3	-306.2	100.5	235.2	234.7	-469.9	57.4	57.4	186.8
CP(PPP)	-18.8	-19.1	-580.3	-303.1	97.0	228.9	228.5	-457.4	55.4	55.5	180.2
aug-cc-pVTZ	-292.9	314.2	314.6	9.3	102.6	-491.9	245.7	246.2	189.6	59.1	59.1
aug-cc-pwCVTZ	184.7	185.0	-393.5	-107.0	99.1	235.2	235.7	-471.0	56.4	56.3	184.4
cc-pwCVTZ	188.5	188.9	-388.9	-102.8	99.0	234.9	235.3	-470.2	56.4	56.3	184.2
def2-TZVP	-261.8	324.8	325.2	27.1	102.3	-476.8	238.2	238.6	187.9	59.5	59.5
def2-TZVP-uncS	19.5	19.8	-567.5	-278.5	102.4	238.4	238.7	-477.1	59.6	59.5	188.1
aug-cc-pVTZ-Jmod	-5.4	-5.8	-586.3	-298.0	98.8	236.5	236.0	-472.5	56.1	56.2	184.1
Exp.	*68.8*	*68.8*	*586.5*								

Table S7. APDs of A_{33}, A_{iso} and ΔA of **$[Cu(NH_3)_4]^{2+}$** for calculated hyperfine coupling constants obtained with TPSSh functional using different basis sets and compared to experimental values.

Basis set	A_{33}	A_{iso}	ΔA	$APD(A_{33})$	$APD(A_{iso})$	$APD(\Delta A)$
aug-cc-pVTZ-Junc	-580.0	195.1	577.6	1	19	12
aug-cc-pVTZ-J	-589.3	205.7	575.6	0	15	11
CP(PPP)	-580.3	206.1	561.5	1	15	8
aug-cc-pVTZ	314.6	112.0	607.5	46	54	17
aug-cc-pwCVTZ	-393.5	7.9	578.5	33	97	12
cc-pwCVTZ	-388.9	3.8	577.8	34	98	12
def2-TZVP	325.2	129.4	587.0	45	46	13
def2-TZVP-uncS	-567.5	176.1	587.3	3	27	13
aug-cc-pVTZ-Jmod	-586.3	199.2	580.9	0	17	12
Exp.	*586.5*	*241.4*	*517.7*			

Figure S2. Graphical representation of APDs of A_{33}, A_{iso} and ΔA of **$[Cu(NH_3)_4]^{2+}$** for calculated hyperfine coupling constants obtained with TPSSh functional and different basis.

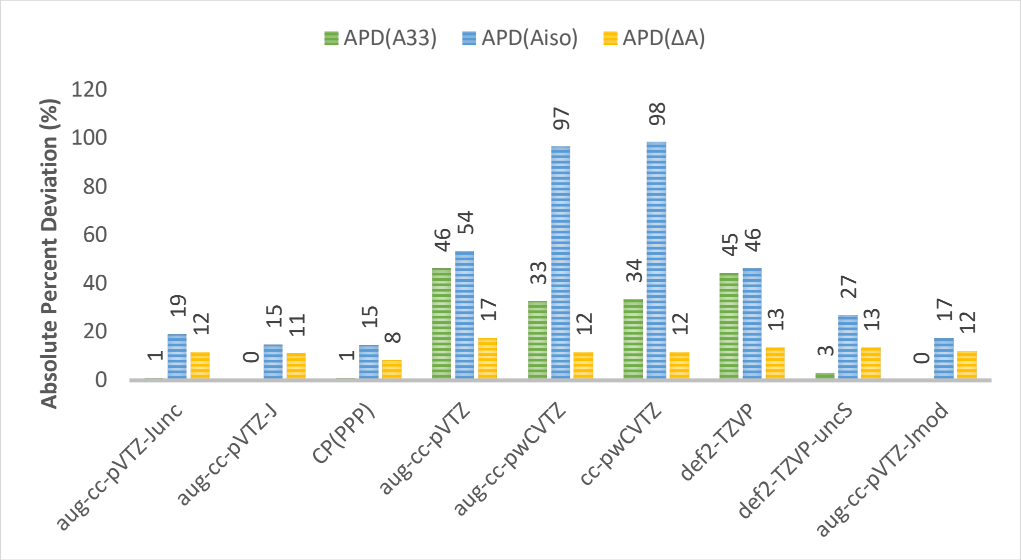

Table S8. Calculated HFCs (individual components and detailed contributions, in MHz) for **[Cu(dtc)$_2$]** obtained with the PBE0 functional with different basis sets.

Basis set	A_{11}	A_{22}	A_{33}	A^{FC}	A^{PC}	A_{11}^{SD}	A_{22}^{SD}	A_{33}^{SD}	$A_{11}^{SO,dip}$	$A_{22}^{SO,dip}$	$A_{33}^{SO,dip}$
aug-cc-pVTZ-Junc	-111.1	-116.7	-528.4	-330.6	78.5	176.1	172.5	-348.7	-35.1	-37.2	72.4
aug-cc-pVTZ-J	-110.1	-115.7	-526.4	-329.9	79.1	176.1	172.5	-348.6	-35.4	-37.4	73.0
CP(PPP)	-111.1	-116.6	-519.1	-324.2	75.3	171.6	167.7	-339.2	-33.7	-35.4	69.1
aug-cc-pVTZ	136.1	141.6	-296.0	-86.9	80.8	180.3	183.8	-364.1	-38.1	-36.1	74.2
aug-cc-pwCVTZ	42.7	48.4	-368.9	-170.6	78.0	172.5	176.1	-348.7	-37.2	-35.2	72.4
cc-pwCVTZ	43.4	49.1	-367.2	-169.3	77.7	172.0	175.7	-347.6	-37.0	-35.0	72.0
def2-TZVP	133.0	138.2	-281.4	-82.0	78.6	173.1	176.2	-349.3	-36.6	-34.7	71.3
def2-TZVP-uncS	-94.6	-99.8	-514.6	-315.1	78.8	176.5	173.3	-349.8	-34.8	-36.7	71.5
aug-cc-pVTZ-Jmod	-109.3	-114.8	-525.5	-329.0	79.1	176.1	172.5	-348.6	-35.4	-37.4	73.0
Exp.	*140.1*	*140.1*	*487.3*								

Table S9. APDs of A_{33}, A_{iso} and ΔA of **[Cu(dtc)$_2$]** for calculated hyperfine coupling constants obtained with PBE0 functional using different basis sets and compared to experimental values.

Basis set	A_{33}	A_{iso}	ΔA	*APD(A_{33})*	*APD(A_{iso})*	*APD(ΔA)*
aug-cc-pVTZ-Junc	-528.4	252.1	417.3	8	1	20
aug-cc-pVTZ-J	-526.4	250.7	416.3	8	2	20
CP(PPP)	-519.1	248.9	408.0	7	3	18
aug-cc-pVTZ	-296.0	6.1	437.6	39	98	26
aug-cc-pwCVTZ	-368.9	92.6	417.3	24	64	20
cc-pwCVTZ	-367.2	91.6	416.3	25	64	20
def2-TZVP	-281.4	3.4	419.6	42	99	21
def2-TZVP-uncS	-514.6	236.3	420.0	6	8	21
aug-cc-pVTZ-Jmod	-525.5	249.9	416.2	8	2	20
Exp.	*487.3*	*255.8*	*347.2*			

Figure S3. Graphical representation of APDs of A_{33}, A_{iso} and ΔA of **[Cu(dtc)$_2$]** for calculated hyperfine coupling constants obtained with PBE0 functional and different basis sets.

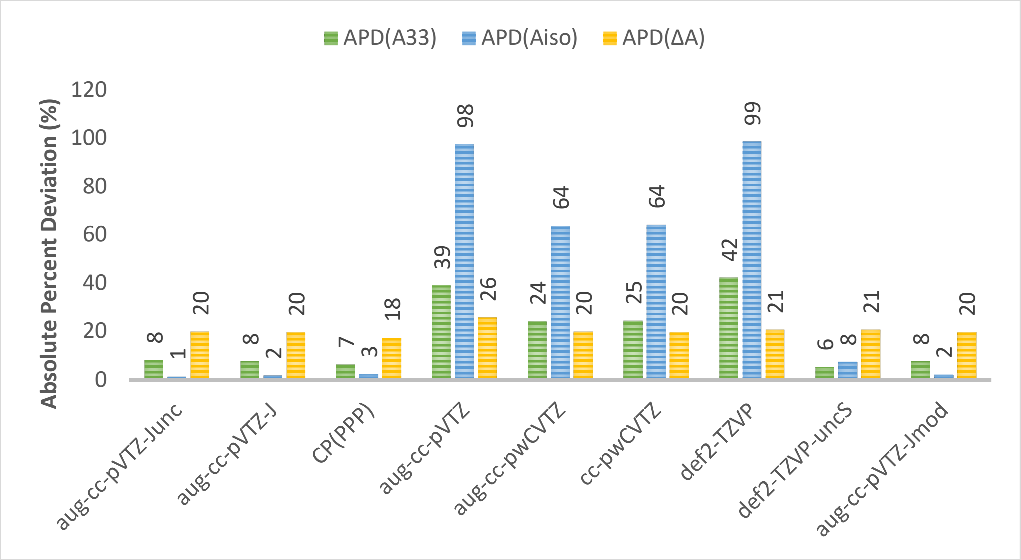

Table S10. Calculated HFCs (individual components and detailed contributions, in MHz) for **[Cu(dtc)$_2$]** obtained with the B3PW91 functional with different basis sets.

Basis set	A_{11}	A_{22}	A_{33}	A^{FC}	A^{PC}	A_{11}^{SD}	A_{22}^{SD}	A_{33}^{SD}	$A_{11}^{SO,dip}$	$A_{22}^{SO,dip}$	$A_{33}^{SO,dip}$
aug-cc-pVTZ-Junc	-98.9	-104.6	-495.3	-302.7	69.7	165.2	161.7	-326.9	-31.2	-33.3	64.6
aug-cc-pVTZ-J	-97.9	-103.5	-493.2	-301.9	70.3	165.1	161.7	-326.8	-31.5	-33.6	65.2
CP(PPP)	-99.4	-104.9	-487.7	-297.8	67.1	161.3	157.5	-318.8	-30.0	-31.8	61.8
aug-cc-pVTZ	128.1	133.7	-281.0	-78.0	71.6	168.6	172.1	-340.7	-34.1	-32.0	66.1
aug-cc-pwCVTZ	47.5	53.1	-342.9	-150.1	69.3	161.6	165.2	-326.8	-33.3	-31.2	64.7
cc-pwCVTZ	48.2	53.9	-341.4	-148.9	69.1	161.2	164.8	-326.0	-33.2	-31.2	64.4
def2-TZVP	126.6	131.8	-266.0	-72.1	69.6	161.9	165.1	-327.0	-32.8	-30.7	63.5
def2-TZVP-uncS	-84.3	-89.5	-482.5	-288.6	69.8	165.3	162.1	-327.4	-30.8	-32.9	63.6
aug-cc-pVTZ-Jmod	-97.3	-102.9	-492.6	-301.2	70.3	165.1	161.7	-326.8	-31.5	-33.6	65.2
Exp.	*140.1*	*140.1*	*487.3*								

Table S11. APDs of A_{33}, A_{iso} and ΔA of **[Cu(dtc)$_2$]** for calculated hyperfine coupling constants obtained with the B3PW91 functional using different basis sets and compared to experimental values.

Basis set	A_{33}	A_{iso}	*ΔA*	*APD(A_{33})*	*APD(A_{iso})*	*APD(ΔA)*
aug-cc-pVTZ-Junc	-495.3	232.9	396.4	2	9	14
aug-cc-pVTZ-J	-493.2	231.5	395.3	1	9	14
CP(PPP)	-487.7	230.7	388.3	0	10	12
aug-cc-pVTZ	-281	6.4	414.7	42	97	19
aug-cc-pwCVTZ	-342.9	80.8	396.0	30	68	14
cc-pwCVTZ	-341.4	79.8	395.3	30	69	14
def2-TZVP	-266	2.5	397.8	45	99	15
def2-TZVP-uncS	-482.5	218.8	398.2	1	14	15
aug-cc-pVTZ-Jmod	-492.6	230.9	395.3	1	10	14
Exp.	*487.3*	*255.8*	*347.2*			

Figure S4. Graphical representation of APDs of A_{33}, A_{iso} and ΔA of **[Cu(dtc)$_2$]** for calculated hyperfine coupling constants obtained with the B3PW91 functional and different basis.

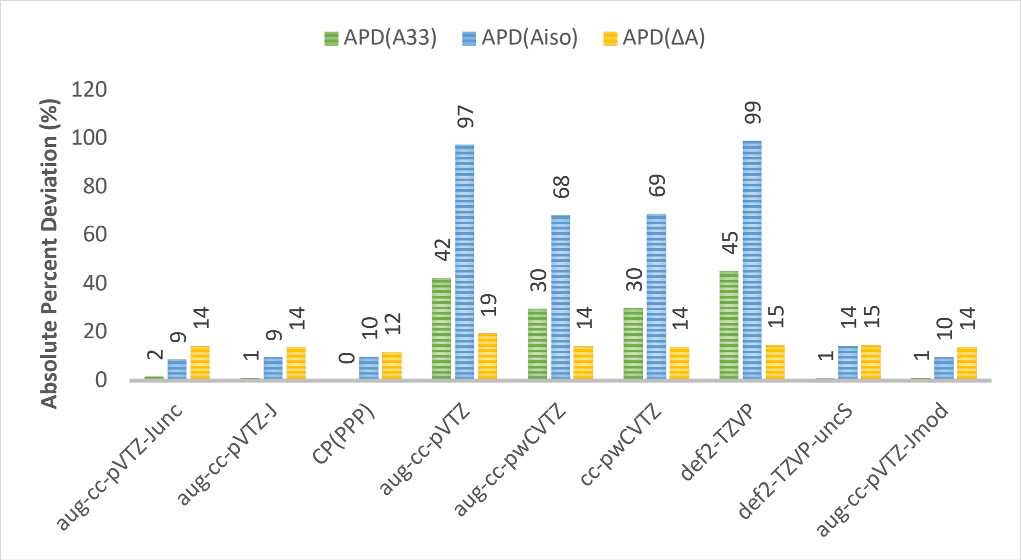

Table S12. Calculated HFCs (individual components and detailed contributions, in MHz) for **[Cu(dtc)$_2$]** obtained with the TPSSh functional with different basis sets.

Basis set	A_{11}	A_{22}	A_{33}	A^{FC}	A^{PC}	A_{11}^{SD}	A_{22}^{SD}	A_{33}^{SD}	$A_{11}^{SO,dip}$	$A_{22}^{SO,dip}$	$A_{33}^{SO,dip}$
aug-cc-pVTZ-Junc	-70.2	-74.5	-463.7	-261.5	58.7	158.1	155.4	-313.5	-25.5	-27.1	52.5
aug-cc-pVTZ-J	-77.6	-81.9	-469.7	-268.9	59.2	157.8	155.1	-312.9	-25.7	-27.3	53.0
CP(PPP)	-75.8	-80.1	-461.8	-263.2	57.3	154.9	151.9	-306.8	-24.8	-26.1	50.9
aug-cc-pVTZ	135.3	139.6	-272.9	-59.7	60.3	162.3	165.0	-327.3	-27.7	-26.1	53.8
aug-cc-pwCVTZ	42.8	47.2	-347.0	-143.8	58.1	155.5	158.3	-313.9	-27.0	-25.4	52.6
cc-pwCVTZ	45.8	50.2	-343.3	-140.5	58.0	155.2	158.1	-313.3	-26.9	-25.4	52.4
def2-TZVP	138.2	142.4	-253.9	-50.4	59.2	156.3	158.8	-315.1	-26.9	-25.3	52.3
def2-TZVP-uncS	-52.2	-56.3	-448.9	-245.2	59.4	159.0	156.4	-315.4	-25.4	-27.0	52.3
aug-cc-pVTZ-Jmod	-69.8	-74.1	-461.9	-261.1	59.2	157.8	155.1	-312.9	-25.7	-27.3	53.0
Exp.	*140.1*	*140.1*	*487.3*								

Table S13. APDs of A_{33}, A_{iso} and ΔA of **[Cu(dtc)$_2$]** for calculated hyperfine coupling constants obtained with the TPSSh functional using different basis sets and compared to experimental values.

Basis set	A_{33}	A_{iso}	***ΔA***	***APD(A_{33})***	***APD(A_{iso})***	***APD(ΔA)***
aug-cc-pVTZ-Junc	-463.7	202.8	393.5	5	21	13
aug-cc-pVTZ-J	-469.7	209.7	392.1	4	18	13
CP(PPP)	-461.8	205.9	386.0	5	20	11
aug-cc-pVTZ	-272.9	0.7	412.5	44	100	19
aug-cc-pwCVTZ	-347	85.7	394.2	29	67	14
cc-pwCVTZ	-343.3	82.4	393.5	30	68	13
def2-TZVP	-253.9	8.9	396.3	48	97	14
def2-TZVP-uncS	-448.9	185.8	396.7	8	27	14
aug-cc-pVTZ-Jmod	-461.9	201.9	392.1	5	21	13
Exp.	*487.3*	*255.8*	*347.2*			

Figure S5. Graphical representation of absolute percent deviation of A_{33}, A_{iso} and ΔA of **[Cu(dtc)$_2$]** for calculated hyperfine coupling constants obtained with the TPSSh functional and different basis sets.

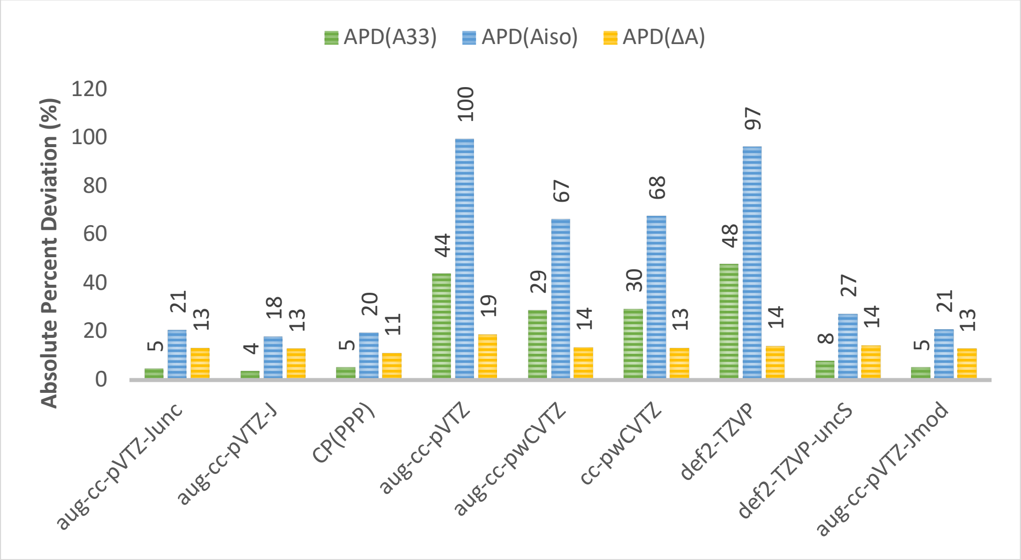

Table S14. Calculated HFCs (individual components and detailed contributions, in MHz) for **[Cu(acac)$_2$]** obtained with the PBE0 functional with different basis sets.

Basis set	A_{11}	A_{22}	A_{33}	A^{FC}	A^{PC}	A_{11}^{SD}	A_{22}^{SD}	A_{33}^{SD}	$A_{11}^{SO,dip}$	$A_{22}^{SO,dip}$	$A_{33}^{SO,dip}$
aug-cc-pVTZ-Junc	-49.2	-50.8	-644.2	-386.5	138.4	263.4	261.7	-525.1	-64.4	-64.4	129.0
aug-cc-pVTZ-J	-47.8	-49.4	-641.2	-385.7	139.5	263.3	261.8	-525.1	-64.9	-65.0	130.0
CP(PPP)	-58.8	-60.2	-637.2	-384.3	132.2	254.9	252.5	-507.4	-61.6	-60.6	122.3
aug-cc-pVTZ	-307.9	314.0	315.7	-34.8	142.1	-547.1	272.9	274.2	131.9	-66.1	-65.8
aug-cc-pwCVTZ	188.4	190.0	-404.5	-146.4	137.7	261.9	263.0	-524.8	-64.7	-64.3	129.0
cc-pwCVTZ	191.9	193.5	-399.6	-141.9	137.1	260.9	262.2	-523.2	-64.2	-64.0	128.3
def2-TZVP	-288.0	313.8	315.2	-27.4	141.0	-530.5	264.4	266.1	128.9	-64.3	-64.4
def2-TZVP-uncS	-26.4	-27.9	-629.9	-369.3	141.2	266.2	264.6	-530.8	-64.5	-64.4	129.0
aug-cc-pVTZ-Jmod	-46.7	-48.4	-640.1	-384.6	139.5	263.3	261.8	-525.1	-64.9	-65.1	130.0
Exp.	*35.0*	*35.0*	*520.0*								

Table S15. APDs of A_{33}, A_{iso} and ΔA of **[Cu(acac)$_2$]** for calculated hyperfine coupling constants obtained with the PBE0 functional using different basis sets and compared to experimental values.

Basis set	A_{33}	A_{iso}	*ΔA*	*APD(A_{33})*	*APD(A_{iso})*	*APD(ΔA)*
aug-cc-pVTZ-Junc	-644.2	248.1	595.0	24	26	23
aug-cc-pVTZ-J	-641.2	246.1	593.4	23	25	22
CP(PPP)	-637.2	252.1	578.4	23	28	19
aug-cc-pVTZ	315.7	107.3	623.6	39	45	29
aug-cc-pwCVTZ	-404.5	8.7	594.5	22	96	23
cc-pwCVTZ	-399.6	4.7	593.1	23	98	22
def2-TZVP	315.2	113.7	603.2	39	42	24
def2-TZVP-uncS	-629.9	228.1	603.5	21	16	24
aug-cc-pVTZ-Jmod	-640.1	245.1	593.4	23	25	22
Exp.	*520.0*	*196.7*	*485.0*			

Figure S6. Graphical representation of APDs of A_{33}, A_{iso} and ΔA of **[Cu(acac)$_2$]** for calculated hyperfine coupling constants obtained with the PBE0 functional.

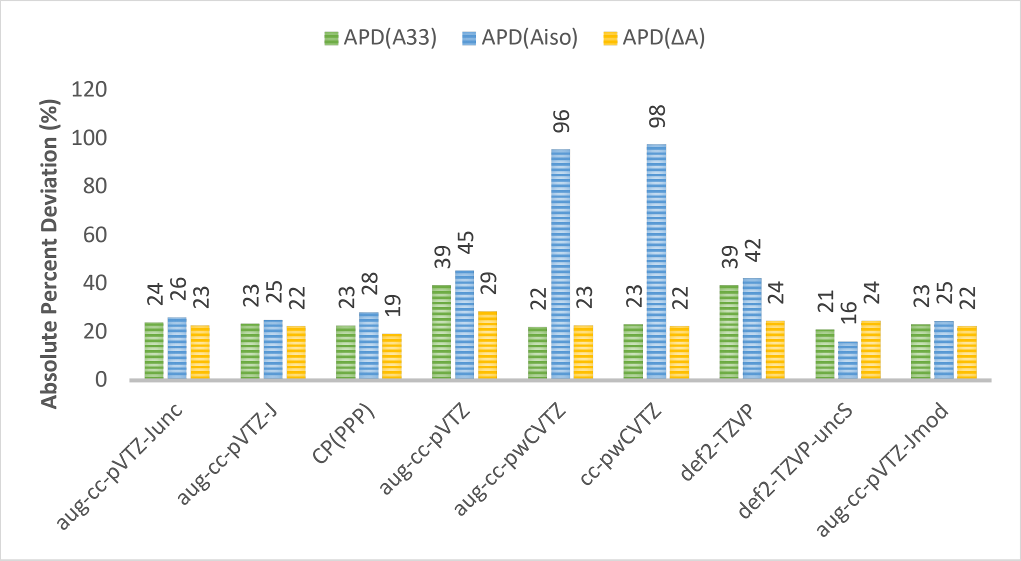

Table S16. Calculated HFCs (individual components and detailed contributions, in MHz) for **[Cu(acac)$_2$]** obtained with the B3PW91 functional with different basis sets.

Basis set	A_{11}	A_{22}	A_{33}	A^{FC}	A^{PC}	A_{11}^{SD}	A_{22}^{SD}	A_{33}^{SD}	$A_{11}^{SO,dip}$	$A_{22}^{SO,dip}$	$A_{33}^{SO,dip}$
aug-cc-pVTZ-Junc	-37.7	-38.1	-622.6	-361.0	128.2	254.2	255.2	-509.4	-59.1	-100.5	119.6
aug-cc-pVTZ-J	-36.1	-36.5	-619.4	-359.9	129.3	254.0	255.4	-509.3	-59.4	-61.2	120.6
CP(PPP)	-48.0	-48.4	-616.5	-359.8	122.2	247.0	245.0	-492.0	-57.4	-55.7	113.2
aug-cc-pVTZ	298.7	300.2	-302.3	-30.6	129.5	259.9	261.6	-521.5	-60.0	-60.4	120.3
aug-cc-pwCVTZ	188.7	190.0	-385.2	-128.0	125.8	249.8	251.2	-501.0	-58.9	-59.0	118.1
cc-pwCVTZ	191.6	192.9	-380.8	-124.1	125.3	248.9	250.5	-499.4	-58.5	-58.8	117.4
def2-TZVP	-281.6	298.7	300.1	-22.0	127.7	-504.1	251.0	253.2	116.8	-58.0	-58.9
def2-TZVP-uncS	-24.1	-25.4	-606.0	-346.3	127.8	253.3	251.1	-504.4	-58.9	-58.0	117.0
aug-cc-pVTZ-Jmod	-40.9	-42.3	-615.4	-360.4	127.5	251.6	249.8	-501.4	-59.7	-59.2	118.9
Exp.	*35.0*	*35.0*	*520.0*								

Table S17. APDs of A_{33}, A_{iso} and ΔA of **[Cu(acac)$_2$]** for calculated hyperfine coupling constants obtained with the B3PW91 functional using different basis sets and compared to experimental values.

Basis set	A_{33}	A_{iso}	***ΔA***	***APD(A_{33})***	***APD(A_{iso})***	***APD(ΔA)***
aug-cc-pVTZ-Junc	-622.6	232.8	584.9	20	18	21
aug-cc-pVTZ-J	-619.4	230.7	583.3	19	17	20
CP(PPP)	-616.5	237.6	568.5	19	21	17
aug-cc-pVTZ	-302.3	98.9	602.5	42	50	24
aug-cc-pwCVTZ	-385.2	2.2	575.2	26	99	19
cc-pwCVTZ	-380.8	1.2	573.7	27	99	18
def2-TZVP	300.1	105.7	581.7	42	46	20
def2-TZVP-uncS	-606	218.5	581.9	17	11	20
aug-cc-pVTZ-Jmod	-615.4	232.9	574.5	18	18	18
Exp.	*520.0*	*196.7*	*485.0*			

Figure S7. Graphical representation of APDs of A_{33}, A_{iso} and ΔA of **[Cu(acac)$_2$]** for calculated hyperfine coupling constants obtained with the B3PW91 functional and different basis sets.

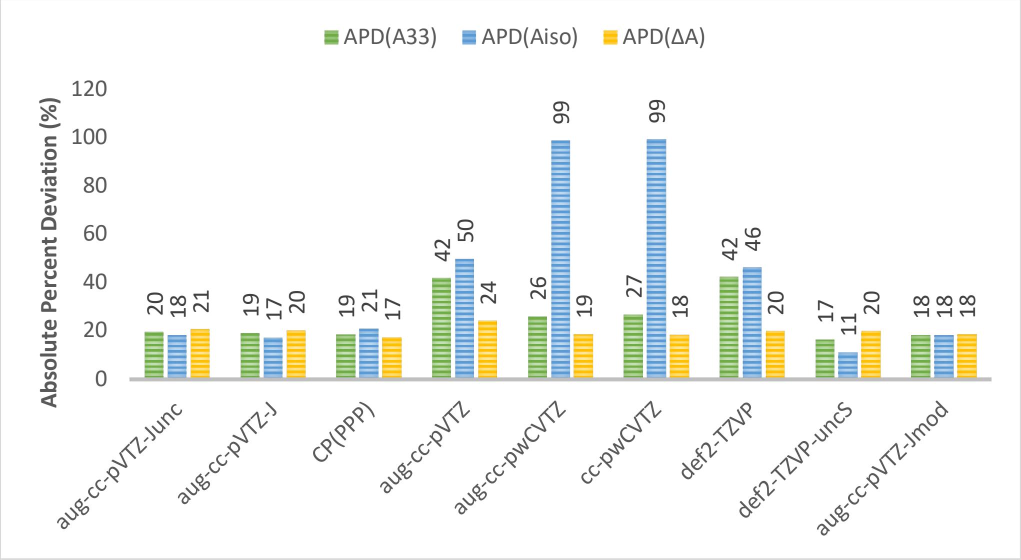

Table S18. Calculated HFCs (individual components and detailed contributions, in MHz) for **[Cu(acac)₂]** obtained with the TPSSh functional with different basis sets.

Basis set	A_{11}	A_{22}	A_{33}	A^{FC}	A^{PC}	A_{11}^{SD}	A_{22}^{SD}	A_{33}^{SD}	$A_{11}^{SO,dip}$	$A_{22}^{SO,dip}$	$A_{33}^{SO,dip}$
aug-cc-pVTZ-Junc	-23.7	-24.5	-606.1	-316.2	98.1	240.4	236.0	-476.5	-46.0	-42.4	88.5
aug-cc-pVTZ-J	-35.1	-36.0	-615.3	-327.8	98.9	240.0	235.7	-475.7	-46.3	-42.8	89.2
CP(PPP)	-42.8	-43.8	-607.0	-325.9	94.7	232.6	228.1	-460.7	-44.3	-40.7	84.9
aug-cc-pVTZ	279.6	281.2	-319.1	-18.9	99.5	242.4	246.5	-488.9	-43.4	-45.9	89.2
aug-cc-pwCVTZ	151.7	153.2	-420.4	-134.7	96.2	232.7	236.6	-469.3	-42.4	-44.9	87.3
cc-pwCVTZ	157.5	159.0	-412.7	-127.9	95.8	231.8	235.8	-467.6	-42.2	-44.7	87.0
def2-TZVP	281.1	282.6	-295.5	-9.2	98.7	233.9	238.3	-472.2	-42.3	-45.1	87.2
def2-TZVP-uncS	-8.2	-9.7	-586.6	-300.2	98.8	238.4	234.1	-472.5	-45.1	-42.3	87.3
aug-cc-pVTZ-Jmod	-23.2	-24.0	-603.4	-315.8	98.9	240.0	235.7	-475.7	-46.3	-42.8	89.2
Exp.	*35.0*	*35.0*	*520.0*								

Table S19. APDs of A_{33}, A_{iso} and ΔA of **[Cu(acac)₂]** for calculated hyperfine coupling constants obtained with the TPSSh functional using different basis sets and compared to experimental values.

Basis set	A_{33}	A_{iso}	*ΔA*	*APD(A_{33})*	*APD(A_{iso})*	*APD(ΔA)*
aug-cc-pVTZ-Junc	-606.1	218.1	582.4	17	11	20
aug-cc-pVTZ-J	-615.3	228.8	580.2	18	16	20
CP(PPP)	-607	231.2	564.2	17	18	16
aug-cc-pVTZ	-319.1	80.6	600.3	39	59	24
aug-cc-pwCVTZ	-420.4	38.5	573.6	19	80	18
cc-pwCVTZ	-412.7	32.1	571.7	21	84	18
def2-TZVP	-295.5	89.4	578.1	43	55	19
def2-TZVP-uncS	-586.6	201.5	578.4	13	2	19
aug-cc-pVTZ-Jmod	-603.4	216.9	580.2	16	10	20
Exp.	*520.0*	*196.7*	*485.0*			

Figure S8. Graphical representation of APDs of A_{33}, A_{iso} and ΔA of **[Cu(acac)₂]** for calculated hyperfine coupling constants obtained with the TPSSh functional and different basis sets.

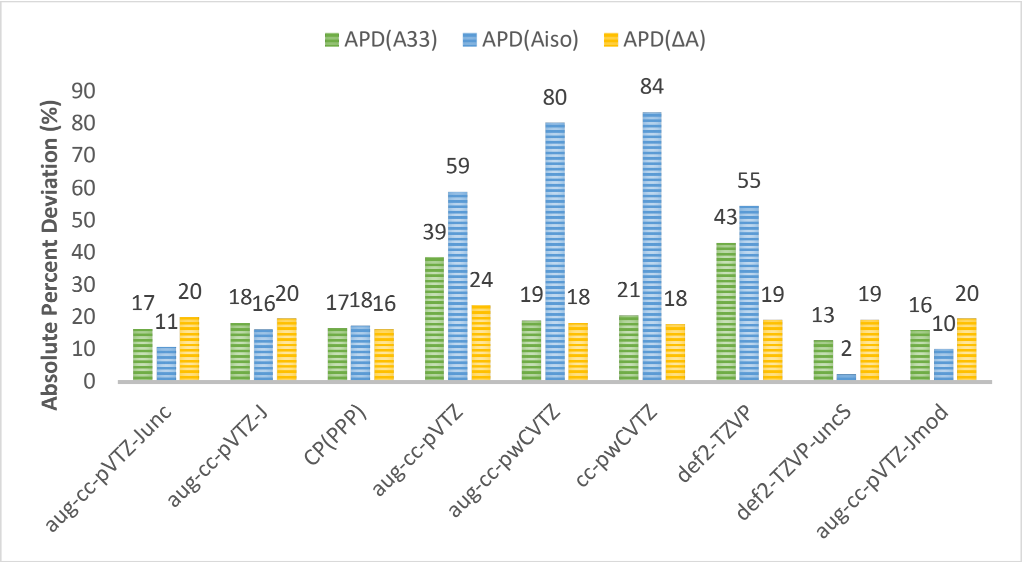

Table S20. Calculated HFCs (individual components and detailed contributions, in MHz) for **$[Cu(NH_3)_4]^{2+}$** obtained with the PBE0 functional and the DKH2 Hamiltonian with different basis sets.

Basis set	A_{11}	A_{22}	A_{33}	A^{FC}	A^{PC}	A_{11}^{SD}	A_{22}^{SD}	A_{33}^{SD}	$A_{11}^{SO,dip}$	$A_{22}^{SO,dip}$	$A_{33}^{SO,dip}$
aug-cc-pVTZ-Junc	-19.6	-20.1	-607.9	-356.4	140.6	258.7	258.1	-516.8	-62.5	-62.4	124.8
aug-cc-pVTZ-J	5.7	6.3	-580.7	-330.8	141.3	257.9	258.5	-516.5	-62.6	-62.7	125.4
CP(PPP)	-24.7	-25.1	-598.1	-351.0	135.0	250.7	250.3	-501.0	-59.5	-59.4	118.9
aug-cc-pVTZ-DK	-295.5	323.9	324.4	-27.0	144.6	-540.3	269.8	270.4	127.2	-63.6	-63.7
aug-cc-pwCVTZ-DK	194.3	194.7	-393.7	-141.4	139.9	258.1	258.6	-516.7	-62.3	-62.4	124.5
cc-pwCVTZ-DK	195.7	196.2	-391.8	-139.7	139.7	257.4	258.3	-516.0	-62.0	-62.1	124.2
DKH-def2-TZVP	-18.9	-19.4	-606.2	-353.2	138.4	257.3	256.8	-514.1	-61.4	-61.3	122.8
def2-TZVP-uncS	4.2	4.7	-596.9	-340.5	144.4	262.7	263.3	-526.0	-62.5	-62.6	125.2
aug-cc-pVTZ-Jmod	-17.8	-18.3	-604.7	-354.9	141.3	258.5	257.9	-516.5	-62.7	-62.6	125.4
Exp.	*68.8*	*68.8*	*586.5*								

Table S21. Calculated HFCs (individual components and detailed contributions, in MHz) for **$[Cu(NH_3)_4]^{2+}$** obtained with the B3PW91 functional, DKH2 Hamiltonian with different basis sets.

Basis set	A_{11}	A_{22}	A_{33}	A^{FC}	A^{PC}	A_{11}^{SD}	A_{22}^{SD}	A_{33}^{SD}	$A_{11}^{SO,dip}$	$A_{22}^{SO,dip}$	$A_{33}^{SO,dip}$
aug-cc-pVTZ-Junc	-13.5	-14.0	-588.9	-335.0	129.5	249.4	248.9	-498.3	-57.4	-57.3	114.9
aug-cc-pVTZ-J	10.5	11.0	-562.9	-310.8	130.3	248.7	249.2	-497.9	-57.7	-57.7	115.4
CP(PPP)	-19.5	-19.8	-579.7	-330.3	124.0	241.4	241.0	-482.4	-54.6	-54.5	109.1
aug-cc-pVTZ-DK	-291.2	313.4	313.8	-21.0	133.0	-520.0	259.7	260.2	116.8	-58.3	-58.4
aug-cc-pwCVTZ-DK	198.0	198.5	-376.6	-122.4	129.1	248.7	249.2	-498.0	-57.3	-57.4	114.7
cc-pwCVTZ-DK	199.0	199.4	-375.2	-121.2	128.9	248.4	248.9	-497.3	-57.2	-57.2	114.4
DKH-def2-TZVP	-15.1	-15.5	-588.5	-332.9	126.5	247.4	246.9	-494.3	-56.1	-56.0	112.2
def2-TZVP-uncS	6.9	7.3	-578.9	-320.3	132.1	252.2	252.7	-504.9	-57.1	-57.1	114.2
aug-cc-pVTZ-Jmod	-12.1	-12.6	-591.5	-334.1	128.7	250.6	250.0	-500.6	-57.3	-57.2	114.6
Exp.	*68.8*	*68.8*	*586.5*								

Table S22. Calculated HFCs (individual components and detailed contributions, in MHz) for **$[Cu(NH_3)_4]^{2+}$** obtained with the PBE0 functional, DKH2 Hamiltonian, and finite nucleus model with different basis sets.

Basis set	A_{11}	A_{22}	A_{33}	A^{FC}	A^{PC}	A_{11}^{SD}	A_{22}^{SD}	A_{33}^{SD}	$A_{11}^{SO,dip}$	$A_{22}^{SO,dip}$	$A_{33}^{SO,dip}$
aug-cc-pVTZ-Junc	-4.5	-5.0	-592.7	-341.3	140.6	258.7	258.1	-516.8	-62.5	-62.4	124.8
aug-cc-pVTZ-J	6.5	7.1	-579.9	-330.1	141.3	257.9	258.5	-516.5	-62.6	-62.7	125.4
CP(PPP)	-22.9	-23.3	-596.2	-349.2	135.0	250.7	250.3	-501.0	-59.5	-59.4	118.9
aug-cc-pVTZ-DK	-295.5	323.8	324.3	-27.0	144.6	-540.3	269.8	270.5	127.2	-63.6	-63.7
aug-cc-pwCVTZ-DK	194.5	195.0	-393.5	-141.2	139.9	258.1	258.6	-516.7	-62.3	-62.4	124.5
cc-pwCVTZ-DK	195.9	196.4	-391.5	-139.4	139.7	257.7	258.3	-516.0	-62.0	-62.1	124.3
DKH-def2-TZVP	-18.8	-19.2	-606.0	-353.1	138.4	257.3	256.8	-514.1	-61.4	-61.3	122.8
def2-TZVP-uncS	4.4	4.9	-596.8	-340.3	144.4	262.7	263.3	-526.0	-62.5	-62.6	125.2
aug-cc-pVTZ-Jmod	-11.0	-11.5	-603.4	-348.3	139.7	260.0	259.4	-519.3	-62.3	-62.2	124.5
Exp.	*68.8*	*68.8*	*586.5*								

Table S23. Calculated HFCs (individual components and detailed contributions, in MHz) for **$[Cu(NH_3)_4]^{2+}$** obtained with the B3PW91 functional, DKH2 Hamiltonian, and finite nucleus model with different basis sets.

Basis set	A_{11}	A_{22}	A_{33}	A^{FC}	A^{PC}	A_{11}^{SD}	A_{22}^{SD}	A_{33}^{SD}	$A_{11}^{SO,dip}$	$A_{22}^{SO,dip}$	$A_{33}^{SO,dip}$
aug-cc-pVTZ-Junc	0.4	0.9	-574.5	-320.6	129.5	248.9	249.4	-498.3	-57.3	-57.4	114.9
aug-cc-pVTZ-J	11.3	11.8	-562.2	-310.0	130.3	248.7	249.2	-497.9	-57.7	-57.7	115.4
CP(PPP)	-17.7	-18.1	-577.9	-328.6	124.0	241.4	241.0	-482.4	-54.6	-54.5	109.1
aug-cc-pVTZ-DK	-291.2	313.3	313.8	-21.1	133.0	-520.0	259.7	260.2	116.8	-58.3	-58.4
aug-cc-pwCVTZ-DK	198.2	198.7	-376.4	-122.3	129.1	248.7	249.2	-498.0	-57.3	-57.4	114.7
cc-pwCVTZ-DK	199.1	199.6	-375.0	-121.0	128.9	248.4	248.9	-497.3	-57.2	-57.2	114.4
DKH-def2-TZVP	-14.9	-15.4	-588.3	-332.7	126.5	247.4	246.9	-494.3	-56.1	-56.0	112.2
def2-TZVP-uncS	7.1	7.5	-578.7	-320.1	132.1	252.2	252.7	-504.9	-57.1	-57.2	114.2
aug-cc-pVTZ-Jmod	-5.2	-5.6	-584.5	-327.2	128.7	250.6	250.0	-500.6	-57.3	-57.2	114.6
Exp.	*68.8*	*68.8*	*586.5*								

Table S24. Calculated HFCs (individual components and detailed contributions, in MHz) for **$[Cu(NH_3)_4]^{2+}$** obtained with the PBE0 functional and ZORA Hamiltonian with different basis sets.

Basis set	A_{11}	A_{22}	A_{33}	A^{FC}	A^{PC}	A_{11}^{SD}	A_{22}^{SD}	A_{33}^{SD}	$A_{11}^{SO,dip}$	$A_{22}^{SO,dip}$	$A_{33}^{SO,dip}$
aug-cc-pVTZ-Junc	-66.5	-67.0	-655.7	-403.4	140.4	258.9	258.4	-517.2	-62.4	-62.3	124.6
aug-cc-pVTZ-J	-41.9	-42.3	-629.8	-379.1	141.1	258.8	258.2	-517.0	-62.6	-62.5	125.2
CP(PPP)	-68.9	69.2	-643.2	-395.3	134.8	251.0	250.5	-501.5	-59.4	-59.3	118.7
aug-cc-pVTZ	-281.5	340.0	340.4	-12.1	145.1	-542.2	270.8	271.3	127.7	-63.8	-63.9
aug-cc-pwCVTZ	234.4	234.8	-356.0	-101.6	139.3	258.7	259.2	-517.8	-62.0	-62.0	124.1
cc-pwCVTZ	237.0	237.4	-353.0	-98.7	139.2	258.3	258.8	-517.1	-61.8	-61.9	123.7
ZORA-def2-TZVP	-58.1	-58.5	-646.2	-392.3	138.0	257.4	257.0	-514.4	-61.3	-61.2	122.4
def2-TZVP-uncS	-30.9	-31.3	-633.5	-376.2	144.4	263.6	263.1	-526.6	-62.6	-62.5	125.0
aug-cc-pVTZ-Jmod	-63.0	-63.5	-651.0	-400.3	141.1	258.8	258.2	-517.0	-62.6	-62.5	125.2
Exp.	*68.8*	*68.8*	*586.5*								

Table S25. Calculated HFCs (individual components and detailed contributions, in MHz) for **$[Cu(NH_3)_4]^{2+}$** obtained with the B3PW91 functional and ZORA Hamiltonian with different basis sets.

Basis set	A_{11}	A_{22}	A_{33}	A^{FC}	A^{PC}	A_{11}^{SD}	A_{22}^{SD}	A_{33}^{SD}	$A_{11}^{SO,dip}$	$A_{22}^{SO,dip}$	$A_{33}^{SO,dip}$
aug-cc-pVTZ-Junc	-57.4	-57.8	-633.6	-379.0	129.4	249.6	249.1	-498.7	-57.4	-57.3	114.7
aug-cc-pVTZ-J	-34.0	-34.4	-608.9	-355.9	130.1	249.4	249.0	-498.4	-57.7	-57.6	115.2
CP(PPP)	-60.9	-61.2	-622.0	-371.8	123.8	241.6	241.3	-482.9	-54.5	-54.4	108.9
aug-cc-pVTZ	-278.6	328.0	328.3	-7.6	133.5	-521.7	260.6	261.1	117.2	-58.6	-58.7
aug-cc-pwCVTZ	235.7	236.1	-341.3	-85.1	128.6	249.3	249.7	-499.0	-57.1	-57.2	114.2
cc-pwCVTZ	237.7	238.1	-338.8	-82.7	128.4	249.0	249.4	-498.4	-56.9	-57.0	113.9
ZORA-def2-TZVP	-51.9	-52.2	626.1	-369.5	126.1	247.5	247.1	-494.5	-56.0	-55.9	111.8
def2-TZVP-uncS	-1.0	-1.4	-580.4	-325.0	130.7	250.1	249.6	-499.8	-56.9	-56.8	113.7
aug-cc-pVTZ-Jmod	-54.1	-54.4	-629.0	-376.0	130.1	249.4	249.0	-498.4	-57.7	-57.6	115.2
Exp.	*68.8*	*68.8*	*586.5*								

Table S26. APDs of A_{33}, A_{iso} and ΔA of **$[Cu(NH_3)_4]^{2+}$** for calculated hyperfine coupling constants obtained with the PBE0 and B3PW91 functionals, using the DKH2 and ZORA Hamiltonians and the aug-cc-pVTZ-J-mod basis set.

Functional	***Relativistic Method***	A_{33}	A_{iso}	***ΔA***	***APD(A_{33})***	***APD(A_{iso})***	***APD(ΔA)***
PBE0	DKH2	-604.7	-213.6	586.9	3	12	13
	DKH2-FN	-603.4	-208.6	592.4	3	14	14
	ZORA	-651.0	-259.2	588.0	11	7	14
	None	-606.4	-218.2	582.6	3	10	13
	Exp.	*586.5*	*241.4*	*517.7*	-	-	-
B3PW91	DKH2	-591.5	-205.4	579.4	1	15	12
	DKH2-FN	-584.5	-198.4	579.3	0	18	12
	ZORA	-629.0	-245.8	574.9	7	2	11
	None	-591.2	-210.0	572.0	1	13	10
	Exp.	*586.5*	*241.4*	*517.7*	-	-	-

Figure S9. Graphical representation of APDs of A_{33}, A_{iso} and ΔA of **$[Cu(NH_3)_4]^{2+}$** for calculated hyperfine coupling constants obtained with the PBE0 functional, using the DKH2 and ZORA Hamiltonians and the aug-cc-pVTZ-J-mod basis set.

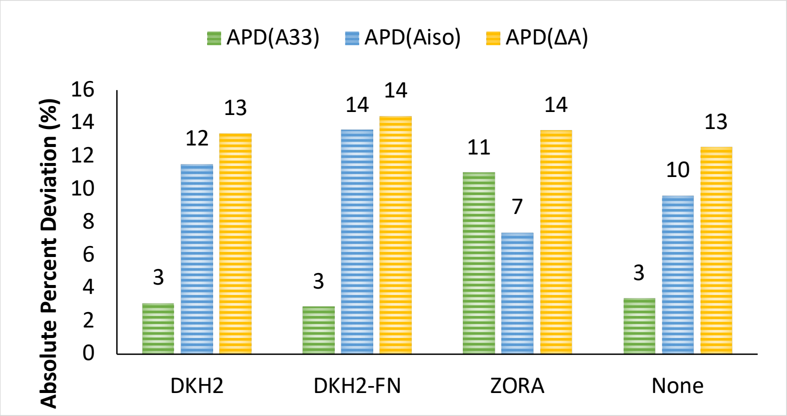

Figure S10. Graphical representation of APDs of A_{33}, A_{iso} and ΔA of **$[Cu(NH_3)_4]^{2+}$** for calculated hyperfine coupling constants obtained with the B3PW91 functional, using the DKH2 and ZORA Hamiltonians and the aug-cc-pVTZ-J-mod basis set.

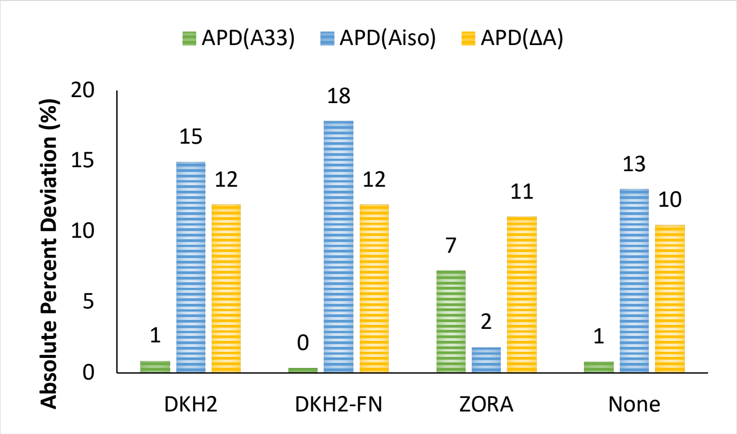

Table S27. Calculated HFCs (individual components, in MHz) and APDs of A_{33}, A_{iso} and ΔA with respect to experimental values for **[Cu(dtc)$_2$]** obtained with the aug-cc-pVTZ-Jmod basis set with different functionals.

Functional	A_{11}	A_{22}	A_{33}	A_{iso}	ΔA	$APD(A_{33})$	$APD(A_{iso})$	$APD(\Delta A)$
B3LYP	-82.7	-88.3	-476.7	215.9	394.0	2.2	15.6	13.5
B3PW91	-97.3	-102.9	-492.6	230.9	395.3	1.1	9.7	13.9
CAM-B3LYP	-121.0	-128.4	-551.1	266.8	430.1	13.1	4.3	23.9
M06	94.5	102.7	-234.0	12.3	336.7	52.0	95.2	3.0
M062X	-257.7	-264.5	-679.8	400.7	422.1	39.5	56.6	21.6
PBE	-41.1	-45.5	-384.0	156.9	342.9	21.2	38.7	1.2
PBE0	-109.3	-114.8	-525.5	249.9	416.2	7.8	2.3	19.9
SCAN	-88.4	-91.5	-516.6	232.2	428.2	6.0	9.3	23.3
TPSS	-41.5	-45.2	-405.7	164.1	364.2	16.7	35.8	4.9
TPSSh	-69.8	-74.1	-461.9	201.9	392.1	5.2	21.1	12.9
Exp.	*140.1*	*140.1*	*487.3*	*255.8*	*347.2*			

Table S28. Calculated HFCs (individual components, in MHz) and APDs of A_{33}, A_{iso} and ΔA with respect to experimental results for **[Cu(acac)$_2$]** obtained with the aug-cc-pVTZ-Jmod basis set with different functionals.

Functional	A_{11}	A_{22}	A_{33}	A_{iso}	ΔA	$APD(A_{33})$	$APD(A_{iso})$	$APD(\Delta A)$
B3LYP	-15.9	-16.3	-605.4	212.5	589.5	16.4	8.1	21.5
B3PW91	-40.9	-42.3	-615.4	232.9	574.5	18.3	18.4	18.5
CAM-B3LYP	-56.3	-59.4	-804.6	306.8	748.3	54.7	56.0	54.3
M06	-133.4	236.0	240.5	114.4	373.9	53.8	41.8	22.9
M062X	-298.6	-306.1	-638.8	414.5	340.2	22.8	110.8	29.9
PBE	-10.4	-10.7	-506.0	175.7	495.6	2.7	10.7	2.2
PBE0	-46.7	-48.4	-640.1	245.1	593.4	23.1	24.6	22.4
SCAN	118.2	233.4	-258.9	30.9	492.3	50.2	84.3	1.5
TPSS	-6.1	-6.8	-537.7	183.5	531.6	3.4	6.7	9.6
TPSSh	-23.2	-24.0	-603.4	216.9	580.2	16.0	10.3	19.6
Exp.	*35.0*	*35.0*	*520.0*	*196.7*	*485.0*			

Table S29. Calculated HFCs (individual components, in MHz) and APDs of A_{33}, A_{iso} and ΔA with respect to experimental results for **[Cu(en)$_2$]$^{2+}$** obtained with the aug-cc-pVTZ-Jmod basis set with different functionals.

Functional	A_{11}	A_{22}	A_{33}	A_{iso}	ΔA	$APD(A_{33})$	$APD(A_{iso})$	$APD(\Delta A)$
B3LYP	-55.3	-56.0	-620.0	243.8	564.7	3.0	4.0	7.8
B3PW91	-72.5	-73.3	-635.0	260.3	562.5	5.5	2.5	7.3
CAM-B3LYP	-60.2	-61.6	-650.9	257.6	590.7	8.1	1.4	12.7
M06	196.2	197.3	-234.1	53.1	431.4	61.1	79.1	17.7
M062X	-296.0	-296.8	-717.6	436.8	421.6	19.2	72.0	19.5
PBE	-27.7	-28.0	-528.3	194.7	500.6	12.2	23.4	4.5
PBE0	-80.7	-81.6	-659.4	273.9	578.7	9.5	7.8	10.4
SCAN	-23.1	-25.2	-654.0	234.1	630.9	8.6	7.8	20.4
TPSS	-26.4	-26.8	-555.3	202.8	528.9	7.8	20.1	0.9
TPSSh	-50.9	-51.5	-613.2	238.5	562.3	1.9	6.1	7.3
Exp.	*78.0*	*82.0*	*602.0*	*254.0*	*524.0*			

Table S30. Calculated HFCs (individual components, in MHz) and APDs of A_{33}, A_{iso} and ΔA with respect to experimental results for **$[Cu(mnt)_2]^{2-}$** obtained with the aug-cc-pVTZ-Jmod basis set with different functionals.

Functional	A_{11}	A_{22}	A_{33}	A_{iso}	*ΔA*	*APD(A_{33})*	*APD(A_{iso})*	*APD(ΔA)*
B3LYP	-66.0	-71.7	-488.1	208.6	422.1	1.1	12.3	14.5
B3PW91	-82.2	-87.5	-503.8	224.5	421.6	4.4	5.6	14.3
CAM-B3LYP	-101.6	-106.2	-559.2	255.7	457.6	15.8	7.5	24.1
M06	125.6	130.7	-252.0	1.4	382.7	47.8	99.4	3.8
M062X	-270.5	-271.5	-766.2	436.1	495.7	58.7	83.3	34.4
PBE	-34.0	-39.8	-390.0	154.6	356.0	19.2	35.0	3.5
PBE0	-95.3	-100.1	-538.3	244.6	443.0	11.5	2.8	20.1
SCAN	19.3	31.6	-129.2	26.1	160.8	73.2	89.0	56.4
TPSS	-35.6	-41.0	-409.9	162.2	374.3	15.1	31.8	1.5
TPSSh	-60.2	-65.4	-468.2	197.9	408.0	3.0	16.8	10.6
Exp.	*113.9*	*116.9*	*482.7*	*237.8*	*368.8*			

Table S31. Calculated HFCs (individual components, in MHz) and APDs of A_{33}, A_{iso} and ΔA with respect to experimental results for **$[Cu(gly)_2]$** obtained with the aug-cc-pVTZ-Jmod basis set with different functionals.

Functional	A_{11}	A_{22}	A_{33}	A_{iso}	*ΔA*	*APD(A_{33})*	*APD(A_{iso})*	*APD(ΔA)*
B3LYP	-27.5	34.4	-581.6	191.6	616.0	9.8	6.7	26.5
B3PW91	16.0	-46.7	-596.5	209.1	612.5	12.6	1.8	25.8
CAM-B3LYP	32.7	-35.5	-618.1	207.0	650.8	16.6	0.8	33.7
M06	-171.6	237.3	281.0	115.6	452.6	47.0	43.7	7.0
M062X	-261.3	-300.5	-703.8	421.9	442.5	32.8	105.4	9.1
PBE	15.8	56.5	-458.5	128.7	515.0	13.5	37.3	5.8
PBE0	5.9	-61.5	-627.0	227.5	632.9	18.3	10.8	30.0
SCAN	13.1	105.4	-581.0	154.2	686.4	9.6	24.9	41.0
TPSS	14.2	61.7	-490.0	138.0	551.7	7.5	32.8	13.3
TPSSh	-21.6	37.1	-567.2	183.9	604.3	7.0	10.5	24.1
Exp.	*43.1*	*43.1*	*529.9*	*205.4*	*486.8*			

Table S32. Calculated HFCs (individual components, in MHz) and APDs of A_{33}, A_{iso} and ΔA with respect to experimental results for **[Cu(kts)]** obtained with the aug-cc-pVTZ-Jmod basis set with different functionals.

Functional	A_{11}	A_{22}	A_{33}	A_{iso}	*ΔA*	*APD(A_{33})*	*APD(A_{iso})*	*APD(ΔA)*
B3LYP	-39.9	-45.1	-548.1	211.0	508.2	4.7	16.8	5.4
B3PW91	-55.9	-61.3	-562.3	226.5	506.4	2.2	10.7	5.0
CAM-B3LYP	-96.0	-101.1	-725.6	307.6	629.6	26.2	21.3	30.6
M06	192.1	200.7	-212.7	60.0	413.4	63.0	76.3	14.3
M062X	-247.6	-254.0	-683.9	395.2	436.3	18.9	55.8	9.5
PBE	-15.1	-20.2	-445.2	160.2	430.1	22.6	36.9	10.8
PBE0	-62.9	-68.3	-592.3	241.2	529.4	3.0	4.9	9.8
SCAN	-11.1	-15.8	-558.5	195.1	547.4	2.9	23.1	13.5
TPSS	-9.5	-14.0	-467.2	163.6	457.7	18.8	35.5	5.1
TPSSh	-32.2	-36.8	-530.6	199.9	498.4	7.7	21.2	3.4
Exp.	*92.9*	*92.9*	*575.1*	*253.6*	*482.2*			

Table S33. Calculated HFCs (individual components, in MHz) and APDs of A_{33}, A_{iso} and ΔA with respect to experimental results for **[Cu(sac)$_2$]** obtained with the aug-cc-pVTZ-Jmod basis set with different functionals.

Functional	A_{11}	A_{22}	A_{33}	A_{iso}	*ΔA*	*APD(A_{33})*	*APD(A_{iso})*	*APD(ΔA)*
B3LYP	37.4	49.9	-566.6	159.8	616.5	20.4	18.9	50.1
B3PW91	16.7	32.0	-581.1	177.5	613.1	23.5	9.9	49.3
CAM-B3LYP	39.1	56.6	-591.5	165.3	648.1	25.7	16.1	57.8
M06	-162.4	299.1	315.9	150.9	478.3	32.9	23.4	16.5
M062X	-237.1	-271.1	-730.4	412.9	493.3	55.2	109.7	20.1
PBE	42.4	50.0	-474.3	127.3	524.3	0.8	35.3	27.7
PBE0	5.4	26.3	-607.0	191.8	633.3	29.0	2.6	54.2
SCAN	102.8	135.0	-252.1	4.8	387.1	46.4	97.6	5.7
TPSS	49.7	54.0	-499.4	131.9	553.4	6.1	33.0	34.7
TPSSh	33.2	39.7	-560.3	162.5	600.0	19.0	17.5	46.1
Exp.	*60.0*	*60.0*	*470.7*	*196.9*	*410.7*			

Table S34. Calculated HFCs (individual components, in MHz) and APDs of A_{33}, A_{iso} and ΔA with respect to experimental results for **[Cu(im)$_4$]$^{2+}$** obtained with the aug-cc-pVTZ-Jmod basis set with different functionals.

Functional	A_{11}	A_{22}	A_{33}	A_{iso}	*ΔA*	*APD(A_{33})*	*APD(A_{iso})*	*APD(ΔA)*
B3LYP	-6.7	-7.5	-610.9	208.4	604.2	8.4	9.4	20.7
B3PW91	-23.9	-24.6	-624.2	224.2	600.3	10.7	2.5	19.9
CAM-B3LYP	-10.2	-11.0	-636.0	219.1	625.8	12.8	4.8	25.0
M06	-181.8	254.8	256.3	109.8	438.1	54.5	52.3	12.5
M062X	-285.1	-287.2	-701.4	424.6	416.3	24.4	84.5	16.8
PBE	5.8	6.5	-534.1	173.9	540.6	5.3	24.4	8.0
PBE0	-33.1	-33.9	-646.3	237.8	613.2	14.6	3.3	22.5
SCAN	23.8	24.1	-654.2	202.1	678.3	16.0	12.2	35.5
TPSS	15.4	15.9	-554.8	174.5	570.7	1.6	24.2	14.0
TPSSh	-3.5	-4.1	-607.7	205.1	604.2	7.8	10.9	20.7
Exp.	*63.2*	*63.2*	*563.8*	*230.1*	*500.6*			

Table S35. Calculated HFCs (individual components, in MHz) and APDs of A_{33}, A_{iso} and ΔA with respect to experimental results for **[Cu(py)$_4$]$^{2+}$** obtained with the aug-cc-pVTZ-Jmod basis set with different functionals.

Functional	A_{11}	A_{22}	A_{33}	A_{iso}	*ΔA*	*APD(A_{33})*	*APD(A_{iso})*	*APD(ΔA)*
B3LYP	-39.7	-40.6	-602.9	227.7	563.2	7.0	3.3	9.4
B3PW91	-55.6	-56.5	-615.0	242.4	559.4	9.1	10.0	8.7
CAM-B3LYP	-43.5	-44.5	-634.2	240.7	590.7	12.5	9.2	14.8
M06	-201.1	209.8	211.5	73.4	412.6	62.5	66.7	19.8
M062X	-294.6	-297.9	-689.4	427.3	394.8	22.3	93.9	23.3
PBE	-19.1	-19.5	-509.7	182.8	490.6	9.5	17.1	4.7
PBE0	-64.1	-65.2	-640.0	256.4	575.9	13.6	16.4	11.9
SCAN	-6.7	-7.4	-631.8	215.3	625.1	12.1	2.3	21.4
TPSS	-11.2	-11.7	-531.8	184.9	520.6	5.6	16.1	1.1
TPSSh	-33.1	-33.7	-591.7	219.5	558.6	5.0	0.4	8.5
Exp.	*48.8*	*48.8*	*563.5*	*220.4*	*514.7*			

Table S36. Calculated HFCs (individual components, in MHz) and APDs of A_{33}, A_{iso} and ΔA with respect to experimental results for **[Cu(eta)]$^{2+}$** obtained with the aug-cc-pVTZ-Jmod basis set with different functionals.

Functional	A_{11}	A_{22}	A_{33}	A_{iso}	*ΔA*	*APD(A_{33})*	*APD(A_{iso})*	*APD(ΔA)*
B3LYP	-44.9	-66.7	-525.4	212.3	480.5	1.2	10.6	8.9
B3PW91	-56.7	-78.8	-537.3	224.3	480.6	1.0	5.6	8.9
CAM-B3LYP	-60.2	-84.0	-588.0	244.1	527.8	10.6	2.7	19.6
M06	141.4	162.9	-231.2	24.4	394.1	56.5	89.7	10.7
M062X	-248.4	-267.9	-688.7	401.7	440.3	29.5	69.1	0.2
PBE	-7.4	-30.0	-401.8	146.4	394.4	24.4	38.4	10.6
PBE0	-65.2	-87.0	-572.1	241.4	506.9	7.6	1.6	14.9
SCAN	-4.3	-52.4	-572.7	209.8	568.4	7.7	11.7	28.8
TPSS	-0.8	-22.8	-422.6	148.7	421.8	20.5	37.4	4.4
TPSSh	-27.3	-48.9	-494.8	190.3	467.5	7.0	19.9	5.9
Exp.	*90.5*	*90.5*	*531.8*	*237.6*	*441.3*			

Table S37. Calculated HFCs (individual components, in MHz) and APDs of A_{33}, A_{iso} and ΔA with respect to experimental results for **[Cu(epa)(H_2O)]$^{2+}$** obtained with the aug-cc-pVTZ-Jmod basis set with different functionals.

Functional	A_{11}	A_{22}	A_{33}	A_{iso}	*ΔA*	*APD(A_{33})*	*APD(A_{iso})*	*APD(ΔA)*
B3LYP	-19.0	-21.9	-588.5	209.8	569.5	12.2	11.4	31.9
B3PW91	-34.9	-38.0	-600.9	224.6	566.0	14.5	5.2	31.1
CAM-B3LYP	-20.3	-23.7	-617.3	220.4	597.0	17.7	6.9	38.3
M06	-202.5	230.4	234.8	87.6	437.3	55.2	63.0	1.3
M062X	-275.6	-279.4	-711.6	422.2	436.0	35.6	78.3	1.0
PBE	-5.8	-7.0	-496.8	169.9	491.0	5.3	28.3	13.7
PBE0	-42.7	-45.8	-626.0	238.2	583.3	19.3	0.6	35.1
SCAN	37.1	40.6	-572.0	164.8	612.6	9.0	30.4	41.9
TPSS	2.6	3.5	-517.8	170.6	521.3	1.3	28.0	20.8
TPSSh	-14.3	-16.9	-576.2	202.5	561.9	9.8	14.5	30.2
Exp.	*92.9*	*92.9*	*524.6*	*236.8*	*431.7*			

Table S38. Calculated HFCs (individual components, in MHz) and APDs of A_{33}, A_{iso} and ΔA with respect to experimental results for **[Cu(atpt)]$^{2+}$** obtained with the aug-cc-pVTZ-Jmod basis set with different functionals.

Functional	A_{11}	A_{22}	A_{33}	A_{iso}	*ΔA*	*APD(A_{33})*	*APD(A_{iso})*	*APD(ΔA)*
B3LYP	-32.4	-46.1	-396.8	158.4	364.4	23.7	10.8	36.6
B3PW91	-40.9	-54.6	-407.1	167.5	366.2	26.9	17.2	37.3
CAM-B3LYP	-54.8	-70.7	-456.8	194.1	402.0	42.4	35.8	50.7
M06	-117.3	136.6	155.0	58.1	272.3	51.7	59.4	2.1
M062X	-176.4	-215.3	-482.6	291.4	306.2	50.4	103.9	14.8
PBE	-5.7	-17.1	-302.6	108.5	296.9	5.7	24.1	11.3
PBE0	-47.2	-61.1	-438.1	182.1	390.9	36.6	27.4	46.5
SCAN	-3.2	0.7	-403.1	135.2	403.8	25.7	5.4	51.3
TPSS	1.6	-8.5	-321.7	109.5	323.3	0.3	23.4	21.2
TPSSh	-18.2	-30.1	-379.1	142.5	360.9	18.2	0.3	35.3
Exp.	*54.0*	*54.0*	*320.8*	*142.9*	*266.8*			

Table S39. Calculated HFCs (individual components, in MHz) and APDs of A_{33}, A_{iso} and ΔA with respect to experimental results for **$[Cu(GGH)_2]^-$** obtained with the aug-cc-pVTZ-Jmod basis set with different functionals.

Functional	A_{11}	A_{22}	A_{33}	A_{iso}	*ΔA*	*APD(A_{33})*	*APD(A_{iso})*	*APD(ΔA)*
B3LYP	-45.8	-52.8	-599.0	232.5	553.2	3.4	7.2	0.1
B3PW91	-64.8	-72.0	-613.5	250.1	548.7	1.0	0.2	1.0
CAM-B3LYP	-38.9	-46.3	-619.6	234.9	580.7	0.1	6.3	4.8
M06	-59.8	212.9	225.2	126.1	285.0	63.7	49.7	48.6
M062X	-252.2	-267.1	-412.1	310.5	159.9	33.5	23.9	71.1
PBE	-27.7	-32.3	-438.1	166.0	410.4	29.3	33.8	25.9
PBE0	-69.1	-76.1	-631.9	259.0	562.8	1.9	3.3	1.6
SCAN	6.6	-32.9	-430.2	152.2	436.8	30.6	39.3	21.2
TPSS	-29.5	-34.4	-511.3	191.7	481.8	17.5	23.5	13.0
TPSSh	-53.5	-59.8	-608.0	240.4	554.5	1.9	4.1	0.1
Exp.	*66.0*	*66.0*	*620.0*	*250.7*	*554.0*			

Table S40. Calculated HFCs (individual components, in MHz) and APDs of A_{33}, A_{iso} and ΔA with respect to experimental results for **$[Cu(GGG)]^-$** obtained with the aug-cc-pVTZ-Jmod basis set with different functionals.

Functional	A_{11}	A_{22}	A_{33}	A_{iso}	*ΔA*	*APD(A_{33})*	*APD(A_{iso})*	*APD(ΔA)*
B3LYP	-30.4	-40.5	-615.7	228.9	585.3	2.2	5.8	8.5
B3PW91	-46.8	-57.0	-628.7	244.2	581.9	4.3	0.5	7.8
CAM-B3LYP	-34.7	-46.2	-651.1	244.0	616.4	8.0	0.5	14.2
M06	206.4	218.9	-268.7	52.2	487.6	55.4	78.5	9.6
M062X	-296.2	-307.5	-810.3	471.3	514.1	34.5	94.1	4.7
PBE	1.1	-12.4	-497.7	169.7	498.8	17.4	30.1	7.6
PBE0	-57.8	-68.2	-658.8	261.6	601.0	9.3	7.7	11.4
SCAN	-6.0	-17.0	-626.0	216.3	620.0	3.9	10.9	14.9
TPSS	4.2	-6.7	-521.1	174.5	525.3	13.5	28.1	2.7
TPSSh	-22.8	-32.3	-592.3	215.8	569.5	1.7	11.1	5.5
Exp.	*63.0*	*63.0*	*602.6*	*242.9*	*539.6*			

Table S41. Calculated HFCs (individual components, in MHz) and APDs of A_{33}, A_{iso} and ΔA with respect to experimental results for **$[Cu(salpn)_2]$** obtained with the aug-cc-pVTZ-Jmod basis set with different functionals.

Functional	A_{11}	A_{22}	A_{33}	A_{iso}	*ΔA*	*APD(A_{33})*	*APD(A_{iso})*	*APD(ΔA)*
B3LYP	3.0	-0.1	-571.1	189.4	574.1	8.4	8.5	19.7
B3PW91	-10.6	-21.7	-584.3	205.5	573.7	10.9	0.7	19.6
CAM-B3LYP	3.7	5.4	-601.7	197.5	607.1	14.2	4.6	26.6
M06	-150.4	245.4	263.5	119.5	413.9	50.0	42.3	13.7
M062X	-254.0	-276.1	-655.5	395.2	401.5	24.5	90.9	16.3
PBE	5.7	6.3	-471.5	153.2	477.8	10.5	26.0	0.4
PBE0	-15.8	-29.3	-610.6	218.6	594.8	15.9	5.6	24.0
SCAN	39.3	47.8	-585.1	166.0	632.9	11.1	19.8	32.0
TPSS	14.3	17.5	-495.0	154.4	512.5	6.0	25.4	6.9
TPSSh	2.1	-2.2	-560.1	186.7	562.2	6.3	9.8	17.2
Exp.	*47.1*	*47.1*	*526.7*	*207.0*	*479.6*			

Table S42. Calculated HFCs (individual components, in MHz) and APDs of A_{33}, A_{iso} and ΔA with respect to experimental results for **[Cu((S,S)-mnpala)$_2$]** obtained with the aug-cc-pVTZ-Jmod basis set with different functionals.

Functional	A_{11}	A_{22}	A_{33}	A_{iso}	*ΔA*	*APD(A_{33})*	*APD(A_{iso})*	*APD(ΔA)*
B3LYP	-49.5	-55.7	-618.5	241.2	569.0	6.3	1.4	10.4
B3PW91	-66.3	-72.5	-631.6	256.8	565.3	8.6	8.0	9.6
CAM-B3LYP	-47.2	-53.8	-650.8	250.6	603.6	11.9	5.4	17.1
M06	187.3	197.6	-218.6	55.4	416.2	62.4	76.7	19.3
M062X	-302.1	-312.3	-705.0	439.8	402.9	21.2	84.9	21.9
PBE	-19.9	-26.6	-501.2	182.6	481.3	13.8	23.2	6.7
PBE0	-74.1	-80.2	-658.8	271.0	584.7	13.3	13.9	13.4
SCAN	-6.9	-14.4	-627.5	216.3	620.6	7.9	9.1	20.4
TPSS	-17.8	-23.9	-530.6	190.8	512.8	8.8	19.8	0.5
TPSSh	-44.9	-50.8	-603.5	233.1	558.6	3.8	2.0	8.3
Exp.	*66.0*	*66.0*	*581.6*	*237.9*	*515.6*			

Table S43. Calculated HFCs (individual components, in MHz) and APDs of A_{33}, A_{iso} and ΔA with respect to experimental results for **[Cu(salen)$_2$]** obtained with the aug-cc-pVTZ-Jmod basis set with different functionals.

Functional	A_{11}	A_{22}	A_{33}	A_{iso}	*ΔA*	*APD(A_{33})*	*APD(A_{iso})*	*APD(ΔA)*
B3LYP	-11.8	-13.9	-609.3	211.7	597.5	0.7	22.0	16.4
B3PW91	-30.3	-32.6	-622.8	228.6	592.5	1.5	15.8	15.4
CAM-B3LYP	-10.0	-12.4	-638.3	220.2	628.3	4.0	18.9	22.4
M06	-210.0	238.6	245.8	91.5	455.8	59.9	66.3	11.2
M062X	-294.3	-304.3	-760.0	452.9	465.7	23.8	66.8	9.3
PBE	-3.3	-4.7	-508.7	172.2	505.4	17.1	36.6	1.5
PBE0	-38.8	-41.2	-649.7	243.2	610.9	5.9	10.4	19.0
SCAN	26.8	26.9	-622.1	189.5	649.0	1.4	30.2	26.4
TPSS	4.1	5.2	-531.8	174.2	537.0	13.3	35.9	4.6
TPSSh	-12.9	-14.0	-596.4	207.8	583.5	2.8	23.5	13.7
Exp.	*100.4*	*100.4*	*613.7*	*271.5*	*513.3*			

Table S44. Calculated HFCs (individual components, in MHz) and APDs of A_{33}, A_{iso} and ΔA with respect to experimental results for **[Cu(bipy)$_2$(NCS)]$^+$** obtained with the aug-cc-pVTZ-Jmod basis set with different functionals.

Functional	A_{11}	A_{22}	A_{33}	A_{iso}	*ΔA*	*APD(A_{33})*	*APD(A_{iso})*	*APD(ΔA)*
B3LYP	47.4	98.4	-475.5	109.9	573.9	1.6	40.7	35.4
B3PW91	31.9	82.8	-487.4	124.2	570.2	4.1	33.0	34.5
CAM-B3LYP	54.2	98.4	-528.0	125.1	626.4	12.8	32.5	47.8
M06	-16.2	300.9	349.2	211.3	365.4	25.4	14.0	13.8
M062X	-141.8	-166.2	-448.5	252.2	306.7	4.2	36.0	27.6
PBE	38.6	75.8	-376.9	87.5	452.7	19.5	52.8	6.8
PBE0	30.0	78.6	-519.9	137.1	598.5	11.1	26.1	41.2
SCAN	63.7	134.9	-501.4	100.9	636.3	7.1	45.6	50.1
TPSS	54.9	94.2	-396.7	82.5	490.9	15.2	55.5	15.8
TPSSh	45.8	92.6	-463.0	108.2	555.6	1.1	41.6	31.1
Exp.	*44.1*	*44.1*	*468.0*	*185.4*	*423.9*			

Table S45. Calculated HFCs (individual components, in MHz) and APDs of A_{33}, A_{iso} and ΔA with respect to experimental results for **$[Cu(ttcn)_2]^{2+}$** obtained with the aug-cc-pVTZ-Jmod basis set with different functionals.

Functional	A_{11}	A_{22}	A_{33}	A_{iso}	*ΔA*	*APD(A_{33})*	*APD(A_{iso})*	*APD(ΔA)*
B3LYP	-18.3	-21.9	-444.0	161.4	425.7	3.2	16.3	6.8
B3PW91	-29.9	-33.8	-456.4	173.4	426.5	0.5	10.1	7.0
CAM-B3LYP	-41.4	-45.2	-509.0	198.5	467.6	11.0	2.9	17.3
M06	170.7	-172.0	178.3	59.0	350.3	61.1	69.4	12.1
M062X	-175.0	-189.4	-572.3	312.2	397.3	24.8	61.9	0.4
PBE	0.6	-2.4	-358.5	120.1	359.1	21.8	37.7	9.9
PBE0	-34.5	-38.7	-485.9	186.4	451.4	5.9	3.4	13.2
SCAN	-17.2	-21.0	-477.0	171.7	459.8	4.0	11.0	15.3
TPSS	9.6	12.5	-372.3	116.7	384.8	18.8	39.5	3.5
TPSSh	-4.4	-7.5	-424.9	145.6	420.5	7.4	24.5	5.5
Exp.	*60.0*	*60.0*	*458.7*	*192.9*	*398.7*			

Table S46. Calculated hyperfine contributions A^{FC} and A^{SD} (in MHz) and their APDs using the aug-cc-pVTZ-Jmod basis set and B2PLYP functional and compared to B3PW91 results.

Complex	*Functional*	A^{FC}	A_{11}^{SD}	A_{22}^{SD}	A_{33}^{SD}	*APD(A^{FC})*	*APD(A_{11}^{SD})*	*APD(A_{22}^{SD})*	*APD(A_{33}^{SD})*
**$[Cu(dtc)_2]^{2+}$**	B2PLYP	-358.9	129.8	122.3	-252.1	19.2	21.4	24.3	22.9
	B3PW91	*-301.2*	*165.1*	*161.7*	*-326.8*				
**$[Cu(acac)_2]$**	B2PLYP	-389.2	278.5	276.0	-554.5	8.0	10.7	10.5	10.6
	B3PW91	*-360.4*	*251.6*	*249.8*	*-501.4*				
**$[Cu(en)_2]^{2+}$**	B2PLYP	*-419.4*	*248.7*	*246.7*	*-495.5*	*13.7*	*4.5*	*4.3*	*4.4*
	B3PW91	*-368.7*	*238.1*	*236.5*	*-474.6*				
**$[Cu(mnt)_2]^{2-}$**	B2PLYP	*-321.9*	*130.6*	*128.1*	*-258.6*	*14.7*	*21.6*	*21.3*	*21.4*
	B3PW91	*-280.7*	*166.5*	*162.7*	*-329.2*				
**$[Cu(gly)_2]$**	B2PLYP	*-371.0*	*309.5*	*217.2*	*-526.6*	*14.1*	*6.5*	*7.6*	*7.0*
	B3PW91	*-325.2*	*290.6*	*201.8*	*-492.4*				
**[Cu(kts)]**	B2PLYP	*-371.8*	*219.2*	*214.4*	*-433.6*	*14.5*	*3.1*	*2.6*	*2.9*
	B3PW91	*-324.9*	*212.5*	*208.9*	*-421.5*				
**$[Cu(sac)_2]$**	B2PLYP	*-319.1*	*275.6*	*259.7*	*-535.3*	*10.2*	*10.5*	*2.3*	*6.4*
	B3PW91	*-289.6*	*249.5*	*253.8*	*-503.3*				
**$[Cu(im)_4]^{2}$**	B2PLYP	*-379.1*	*266.4*	*266.3*	*-532.8*	*11.0*	*4.6*	*4.6*	*4.6*
	B3PW91	*-341.6*	*254.6*	*254.7*	*-509.4*				
**$[Cu(py)_4]^{2+}$**	B2PLYP	*-396.0*	*252.3*	*252.0*	*-504.3*	*11.9*	*5.7*	*5.5*	*5.6*
	B3PW91	*-353.8*	*238.6*	*238.9*	*-477.5*				
**$[Cu(eta)]^{2+}$**	B2PLYP	-386.0	247.5	196.9	-444.4	22.3	19.7	6.1	13.3
	B3PW91	-315.6	206.8	185.5	-392.2				
**$[Cu(epa)(H_2O)]^{2}$**	B2PLYP	-358.4	251.9	247.0	-498.9	7.3	4.9	4.8	4.9
	B3PW91	-334.0	240.1	235.7	-475.7				
**$[Cu(atpt)]^{2+}$**	B2PLYP	-335.6	160.7	145.1	-305.8	29.4	3.2	7.8	5.5
	B3PW91	-259.4	166.0	157.4	-323.4				
**$[Cu(H_{-2}GGH)_2]^{-}$**	B2PLYP	-391.5	229.4	175.8	-405.2	42.7	18.9	13.3	16.4
	B3PW91	-274.4	193.0	155.1	-348.1				
**$[Cu(H_{-2}GGG)]^{-}$**	B2PLYP	-387.2	251.6	239.8	-491.4	12.7	4.3	4.0	4.2
	B3PW91	-343.5	241.3	230.5	-471.8				
**$[Cu(salpn)_2]$**	B2PLYP	-359.0	269.9	254.3	-524.2	10.1	10.6	5.2	7.9
	B3PW91	-326.2	244.0	241.7	-485.7				
**$[Cu(S,S\text{-}mnpala)_2]$**	B2PLYP	-411.9	260.5	255.3	-515.8	11.1	7.2	7.5	7.4

	B3PW91	-370.8	242.9	237.5	-480.4				
**$[Cu(salen)_2]$**	B2PLYP	-372.1	265.7	262.4	-528.0	9.2	8.1	4.7	6.4
	B3PW91	-340.7	245.9	250.5	-496.4				
**$[Cu(bipy)_2(NCS)]^{+}$**	B2PLYP	-304.4	299.1	241.2	-540.3	16.8	44.4	11.2	12.8
	B3PW91	-260.6	207.2	271.7	-478.8				
**$[Cu(ttcn)_2]^{2+}$**	B2PLYP	-325.2	169.2	167.3	-336.6	25.4	5.2	5.8	5.5
	B3PW91	-259.3	178.6	177.6	-356.2				

Table S47. Calculated HFCs (total values, in MHz) using the aug-cc-pVTZ-Jmod basis set and B2PLYP functional, and their APDs with respect to experimental values using the B3PW91 value for the A^{SO} contribution.

Complex	A_{33}	A_{iso}	ΔA	***MAPD(A33)***	***MAPD(Aiso)***	***MAPD(ΔA)***
**$[Cu(NH_3)_4]^{2+}$**	-663.3	254.8	613.0	13.1	5.5	18.4
**$[Cu(dtc)_2]^{2+}$**	-475.6	288.6	285.2	2.4	12.8	17.8
**$[Cu(acac)_2]$**	-697.3	261.7	654.5	34.1	33.1	35.0
**$[Cu(en)_2]^{2+}$**	-706.5	310.9	594.0	17.4	22.4	13.4
**$[Cu(mnt)_2]^{2-}$**	-474.4	265.7	315.2	1.7	11.7	14.5
**$[Cu(gly)_2]$**	-676.5	254.8	665.7	27.7	30.4	28.8
**[Cu(kts)]**	-621.5	273.5	525.3	8.1	7.8	8.9
**$[Cu(sac)_2]$**	-642.6	207.0	655.8	36.5	5.1	59.7
**$[Cu(im)_4]^{2}$**	-685.1	261.7	635.5	21.5	13.7	27.0
**$[Cu(py)_4]^{2+}$**	-683.9	284.5	599.7	21.4	29.1	16.5
**$[Cu(eta)]^{2+}$**	-659.9	294.7	573.5	24.1	24.0	30.0
**$[Cu(epa)(H_2O)]^{2}$**	-648.4	248.9	601.0	23.6	5.1	39.2
**$[Cu(atpt)]^{2+}$**	-465.6	243.7	343.2	45.1	70.5	28.6
**$[Cu(H_{-2}GGH)_2]^{-}$**	-641.0	307.3	525.5	3.4	22.6	5.1
**$[Cu(H_{-2}GGG)]^{-}$**	-692.0	287.9	611.8	14.8	18.5	13.4
**$[Cu(salpn)_2]$**	-655.7	238.4	636.9	24.5	15.2	32.8
**$[Cu(S,S\text{-}mnpala)_2]$**	-708.0	297.9	618.2	21.7	25.2	19.9
**$[Cu(salen)_2]$**	-685.9	260.0	644.0	11.8	4.3	25.5
**$[Cu(bipy)_2(NCS)]^{+}$**	-592.7	168.0	672.8	26.6	9.4	58.7
**$[Cu(ttcn)_2]^{2+}$**	-502.6	239.2	397.5	9.6	24.0	0.3

Table S48. Calculated hyperfine contributions A^{FC} and A^{SD} (in MHz) and their APDs, using the aug-cc-pVTZ-Jmod basis set and wave function based methods, and compared to DFT results using B3PW91 functional.

Complex	*Method*	A^{FC}	A_{11}^{SD}	A_{22}^{SD}	A_{33}^{SD}	*APD(A^{FC})*	*APD(A_{11}^{SD})*	*APD(A_{22}^{SD})*	*APD(A_{33}^{SD})*
**$[Cu(NH_3)_4]^{2+}$**	DLPNO	-376.1	278.5	277.7	-556.1	11	12	12	12
	HF	-467.9	320.0	319.0	-639.0	38	29	29	29
	MP2	-458.5	301.6	300.8	-602.4	36	22	22	22
	OO-MP2	-328.3	160.1	159.9	-320.0	3	35	35	35
	OO-SCS-MP2	-275.8	208.6	208.2	-416.8	18	16	16	16
	B3PW91	*-337.9*	*248.1*	*247.6*	*-495.7*				
**$[Cu(dtc)_2]^{2+}$**	DLPNO	-343.0	201.5	195.6	-397.1	13.9	22.0	21.0	21.5
	HF	-476.9	310.3	309.6	-619.9	58.3	87.9	91.5	89.7
	MP2	-473.6	266.9	260.8	-527.8	57.2	61.6	61.3	61.5
	OO-MP2	-150.1	103.6	90.9	-194.4	50.2	37.3	43.8	40.5
	OO-SCS-MP2	-96.3	115.5	117.9	-233.3	68.0	30.1	27.1	28.6
	B3PW91	*-301.2*	*165.1*	*161.7*	*-326.8*				
**$[Cu(acac)_2]$**	DLPNO	-370.6	294.7	293.1	-587.8	2.8	17.1	17.3	17.2
	HF	-499.7	326.8	324.5	-651.3	38.7	29.9	29.9	29.9
	MP2	-471.1	314.1	312.6	-626.8	30.7	24.8	25.2	25.0
	OO-MP2	-115.7	123.0	128.9	-252.0	67.9	51.1	48.4	49.7
	OO-SCS-MP2	-132.2	185.1	203.0	-388.1	63.3	26.4	18.7	22.6
	B3PW91	*-360.4*	*251.6*	*249.8*	*-501.4*				
**$[Cu(en)_2]^{2+}$**	DLPNO	-393.7	270.1	268.1	-538.2	6.8	13.4	13.3	13.4
	HF	-510.4	320.5	318.7	-639.1	38.4	34.6	34.7	34.7
	MP2	-500.4	298.2	296.1	-594.3	35.7	25.2	25.2	25.2
	OO-MP2	-287.4	152.0	149.2	-301.2	22.1	36.2	36.9	36.5
	OO-SCS-MP2	-225.1	191.8	191.5	-383.3	39.0	19.4	19.1	19.2
	B3PW91	*-368.7*	*238.1*	*236.5*	*-474.6*				
**$[Cu(mnt)_2]^{2-}$**	DLPNO	-343.2	195.8	194.9	-390.7	22.3	17.6	19.8	18.7
	HF	-475.0	304.0	301.5	-605.6	69.2	82.6	85.3	83.9
	MP2	-451.0	237.7	235.7	-473.4	60.7	42.8	44.8	43.8
	OO-MP2	-104.1	84.4	76.1	-160.5	62.9	49.3	53.2	51.3
	OO-SCS-MP2	-123.3	120.5	92.5	-213.0	56.1	27.7	43.1	35.3
	B3PW91	*-280.7*	*166.5*	*162.7*	*-329.2*				
**$[Cu(gly)_2]$**	DLPNO	-355.5	339.0	223.8	-562.8	9.3	16.7	10.9	14.3
	HF	-497.4	401.9	238.0	-639.9	52.9	38.3	17.9	30.0
	MP2	-463.8	357.9	249.1	-607.0	42.6	23.2	23.5	23.3
	OO-MP2	-122.7	122.6	136.9	-259.5	62.3	57.8	32.1	47.3
	OO-SCS-MP2	-132.5	163.6	227.8	-391.4	59.3	43.7	12.9	20.5
	B3PW91	*-325.2*	*290.6*	*201.8*	*-492.4*				
**[Cu(kts)]**	DLPNO	-339.4	250.9	244.7	-495.6	4.5	18.0	17.1	17.6
	HF	-477.8	320.1	313.5	-633.6	47.1	50.6	50.0	50.3
	MP2	-445.9	292.7	284.9	-577.6	37.3	37.7	36.3	37.0
	OO-MP2	-46.6	90.6	102.1	-192.7	85.7	57.4	51.1	54.3
	OO-SCS-MP2	-62.2	123.7	139.8	-263.4	80.9	41.8	33.1	37.5
	B3PW91	*-324.9*	*212.5*	*208.9*	*-421.5*				
**$[Cu(sac)_2]$**	DLPNO	-323.4	298.5	270.2	-568.7	11.7	19.6	6.4	13.0
	HF	-507.3	347.5	295.8	-643.3	75.2	39.3	16.5	27.8
	MP2	149.0	252.9	-646.9	394.0	48.5	1.4	154.9	21.7
	OO-MP2	-95.6	81.9	153.5	-235.4	67.0	67.2	39.5	53.2
	OO-SCS-MP2	-113.1	163.3	207.5	-370.8	60.9	34.6	18.3	26.3
	B3PW91	*-289.6*	*249.5*	*253.8*	*-503.3*				

Complex	***Method***	A^{FC}	A_{11}^{SD}	A_{22}^{SD}	A_{33}^{SD}	***APD***(A^{FC})	***APD***(A_{11}^{SD})	***APD***(A_{22}^{SD})	***APD***(A_{33}^{SD})
**[Cu(im)$_4$]^2**	DLPNO	-379.3	283.2	283.0	-566.2	11.0	11.2	11.1	11.2
	HF	-499.5	321.9	321.5	-643.4	46.2	26.4	26.2	26.3
	MP2	-440.2	304.3	304.2	-608.5	28.9	19.5	19.4	19.5
	OO-MP2	-250.0	155.6	155.4	-311.0	26.8	38.9	39.0	38.9
	OO-SCS-MP2	-230.5	207.1	206.6	-413.7	32.5	18.7	18.9	18.8
	B3PW91	*-341.6*	*254.6*	*254.7*	*-509.4*				
**[Cu(py)$_4$]$^{2+}$**	DLPNO	-375.4	274.7	274.4	-549.1	6.1	15.1	14.9	15.0
	HF	-692.1	315.3	314.8	-630.1	95.6	32.1	31.8	31.9
	MP2	111.7	313.9	314.6	-628.5	68.4	31.5	31.7	31.6
	OO-MP2	-201.6	128.8	128.3	-257.1	43.0	46.0	46.3	46.2
	OO-SCS-MP2	-171.4	176.2	177.1	-353.2	51.5	26.2	25.9	26.0
	B3PW91	*-353.8*	*238.6*	*238.9*	*-477.5*				
**[Cu(eta)]$^{2+}$**	DLPNO	-339.2	275.9	227.9	-503.8	7.5	33.4	22.9	28.5
	HF	-581.4	344.1	280.3	-624.3	84.2	66.4	51.1	59.2
	MP2	-299.8	326.3	264.4	-590.7	5.0	57.8	42.6	50.6
	OO-MP2	-38.2	73.0	138.7	-211.7	87.9	64.7	25.2	46.0
	OO-SCS-MP2	-64.3	112.0	169.2	-281.3	79.6	45.8	8.8	28.3
	B3PW91	*-315.6*	*206.8*	*185.5*	*-392.2*				
**[Cu(epa)(H_2O)]^2**	DLPNO	-353.2	271.0	266.9	-537.9	5.7	12.9	13.3	13.1
	HF	-476.9	320.9	317.9	-638.9	42.8	33.7	34.9	34.3
	MP2	-448.1	312.3	285.2	-597.5	34.2	30.1	21.0	25.6
	OO-MP2	26.8	-34.5	8.4	26.1	92.0	85.6	96.4	94.5
	OO-SCS-MP2	-60.3	81.1	85.5	-166.7	82.0	66.2	63.7	65.0
	B3PW91	*-334.0*	*240.1*	*235.7*	*-475.7*				
**[Cu(atpt)]$^{2+}$**	DLPNO	-302.1	203.0	194.0	-397.0	16.5	22.3	23.3	22.8
	HF	-410.3	308.8	307.4	-616.1	58.2	86.0	95.3	90.5
	MP2	-398.3	278.0	269.5	-547.4	53.5	67.4	71.2	69.3
	OO-MP2	-91.4	76.7	61.4	-138.1	64.7	53.8	61.0	57.3
	OO-SCS-MP2	-60.1	81.9	95.2	-177.1	76.8	50.7	39.5	45.2
	B3PW91	*-259.4*	*166.0*	*157.4*	*-323.4*				
**[Cu(H$_{-2}$GGH)$_2$]$^-$**	DLPNO	-309.1	253.1	219.0	-472.1	12.6	31.2	41.2	35.6
	HF	-446.7	365.4	266.4	-631.8	62.8	89.4	71.8	81.5
	MP2	-423.2	306.9	273.0	-579.9	54.3	59.0	76.0	66.6
	OO-MP2	-38.0	19.9	106.6	-126.4	86.2	89.7	31.3	63.7
	OO-SCS-MP2	11.9	42.6	137.7	-180.4	95.7	77.9	11.2	48.2
	B3PW91	*-274.4*	*193.0*	*155.1*	*-348.1*				
**[Cu(H$_{-2}$GGG)]$^-$**	DLPNO	-365.6	278.3	258.9	-537.2	6.4	15.3	12.3	13.9
	HF	-509.0	350.1	288.5	-638.6	48.2	45.1	25.2	35.4
	MP2	-474.0	305.0	284.1	-589.2	38.0	26.4	23.3	24.9
	OO-MP2	-131.9	132.9	109.3	-242.2	61.6	44.9	52.6	48.7
	OO-SCS-MP2	-127.4	155.1	183.7	-338.9	62.9	35.7	20.3	28.2
	B3PW91	*-343.5*	*241.3*	*230.5*	*-471.8*				
**[Cu(salpn)$_2$]**	DLPNO	-347.6	290.0	271.4	-561.4	6.6	18.9	12.3	15.6
	HF	-512.3	334.2	307.0	-641.2	57.1	37.0	27.0	32.0
	MP2	-18.8	311.1	330.7	-641.8	94.2	27.5	36.8	32.2
	OO-MP2	-82.6	83.5	108.3	-191.9	74.7	65.8	55.2	60.5
	OO-SCS-MP2	-72.3	139.0	167.4	-306.4	77.8	43.0	30.7	36.9
	B3PW91	*-326.2*	*244.0*	*241.7*	*-485.7*				
**[Cu(S,S-mnpala)$_2$]**	DLPNO	-380.0	282.6	277.2	-559.7	2.5	16.3	16.7	16.5
	HF	-511.9	326.1	318.1	-644.2	38.0	34.2	33.9	34.1
	MP2	-486.2	306.4	301.0	-607.5	31.1	26.1	26.8	26.4
	OO-MP2	-145.3	131.0	118.4	-249.4	60.8	46.1	50.1	48.1
	OO-SCS-MP2	-134.6	179.3	185.6	-364.9	63.7	26.2	21.9	24.0
	B3PW91	*-370.8*	*242.9*	*237.5*	*-480.4*				

Complex	***Method***	A^{FC}	A_{11}^{SD}	A_{22}^{SD}	A_{33}^{SD}	***APD***(A^{FC})	***APD***(A_{11}^{SD})	***APD***(A_{22}^{SD})	***APD***(A_{33}^{SD})
**[Cu(salen)$_2$]**	DLPNO	-365.5	283.2	281.3	-564.5	7.3	15.2	12.3	13.7
	HF	-534.2	324.7	318.7	-643.4	56.8	32.1	27.2	29.6
	MP2	-88.8	303.8	330.4	-634.2	73.9	23.6	31.9	27.8
	OO-MP2	-92.9	108.6	115.3	-223.8	72.7	55.8	54.0	54.9
	OO-SCS-MP2	-120.4	160.6	187.2	-347.8	64.7	34.7	25.3	29.9
	B3PW91	*-340.7*	*245.9*	*250.5*	*-496.4*				
**[Cu(bipy)$_2$(NCS)]$^+$**	DLPNO	-307.9	319.3	257.2	-576.5	18.1	54.1	5.3	20.4
	HF	-395.7	361.6	292.2	-653.8	51.8	74.6	7.5	36.5
	MP2	-420.5	319.5	294.3	-613.7	61.3	54.2	8.3	28.2
	OO-MP2	-32.2	33.3	101.3	-134.7	87.6	83.9	62.7	71.9
	OO-SCS-MP2	25.8	58.3	105.6	-163.9	90.1	71.8	61.1	65.8
	B3PW91	*-260.6*	*207.2*	*271.7*	*-478.8*				
**[Cu(ttcn)$_2$]$^{2+}$**	DLPNO	-318.1	222.0	217.0	-439.0	22.7	24.3	22.2	23.3
	HF	-410.4	318.3	312.7	-631.0	58.2	78.2	76.1	77.2
	MP2	-395.5	283.1	278.8	-561.9	52.5	58.5	57.0	57.7
	OO-MP2	-172.0	96.9	94.5	-191.4	33.7	45.8	46.8	46.3
	OO-SCS-MP2	-131.0	115.1	113.3	-228.4	49.5	35.6	36.2	35.9
	B3PW91	*-259.3*	*178.6*	*177.6*	*-356.2*				
